# Hierarchical differentiation and design gaps in China's Internet Plus Nursing Services Policies: a PMC index analysis

**DOI:** 10.3389/fpubh.2026.1829126

**Published:** 2026-05-08

**Authors:** Yuzhe Wang, Yingchun Liu, Chunjie Jin, Fang Hu, Tianzhi Yu

**Affiliations:** 1 School of Management, Tianjin University of Traditional Chinese Medicine, Tianjin, China; 2 Internet Hospital, Tianjin Medical University General Hospital, Tianjin, China; 3 Hospital Administration Office, Tianjin Medical University General Hospital, Tianjin, China

**Keywords:** internet healthcare, internet plus nursing services, multi-level governance, policy evaluation, policy instruments

## Abstract

**Introduction:**

As population aging accelerates and digital health technologies continue to expand, integrating internet-based nursing services into long-term care systems has become an important policy priority. However, existing studies have paid limited attention to how policy themes, instrument configurations, and design consistency interact across governance levels in this field. To address this gap, this study develops a three-dimensional analytical framework encompassing policy themes, policy instruments, and design consistency to examine the structural characteristics and design quality of China's “Internet Plus Nursing Services” policies.

**Methods:**

From a multi-level governance perspective, this study analyzed 123 national and local policy documents on “Internet Plus Nursing Services” issued in China between 2018 and 2025. A three-dimensional analytical framework integrating keyword co-occurrence analysis, Rothwell and Zegveld's policy instrument framework, and the Policy Modeling Consistency (PMC) index model was applied to examine thematic structures, policy tool configurations, and design quality.

**Results:**

National and local policies showed clear hierarchical differentiation in thematic focus, instrument configuration, and design consistency. National policies were more strongly oriented toward regulatory coordination and system governance, whereas local policies placed greater emphasis on service implementation and community-based delivery. Although environment-oriented instruments remained dominant at both levels, structural imbalances persisted, particularly in the alignment between regulatory requirements and incentive arrangements and between supply-side capacity building and demand-side activation. PMC analysis further indicated stronger overall design consistency at the national level than at the local level (7.98 vs. 7.31). Across both levels, policy coverage and evaluation mechanisms remained the weakest dimensions, with local policies showing particularly limited performance in policy coverage (X3 = 0.27), suggesting persistent gaps in service scope, feedback, and adaptive adjustment.

**Conclusions:**

Although “Internet Plus Nursing Services” policies have achieved substantial institutional expansion, important gaps remain in internal coordination and design balance. Future policy development should place greater emphasis on aligning regulation with incentives, improving coordination between supply-side capacity building and demand-side activation, and strengthening policy coverage, evaluation, and feedback mechanisms, especially at the local level. These findings suggest that the quality of digital nursing governance depends not only on policy expansion, but also on the structural coherence of policy arrangements across governance levels. The three-dimensional analytical framework developed in this study provides a replicable approach for assessing digital nursing governance and may offer useful insights for other countries advancing digital transformation in long-term care systems.

## 1 Introduction

As the acceleration of population aging continues and the burden of chronic diseases rises persistently, the demand for long-term and continuous care has increased significantly. The traditional care model centered on medical institutions is under pressure in terms of service accessibility, continuity, and cost control. The WHO's Global Strategy on Digital Health 2020–2025 highlights that the deep integration of digital technologies with primary care systems is a critical pathway for transforming health systems (1). In China, the National Health Commission launched pilot programs for “Internet Plus Nursing Services” in 2019, aiming to integrate nursing resources through online platforms and standardize home-visit procedures to enhance the accessibility and continuity of nursing services (2). The integration of digital technologies into nursing and long-term care systems contributes to improved resource allocation efficiency and strengthened coordination in hierarchical diagnosis and treatment (3, 4). Therefore, “Internet Plus Nursing Services” has gradually become an important institutional tool for advancing the transformation of the nursing service system.

As internet-based healthcare becomes increasingly embedded in health systems, its governance logic and policy structure have attracted growing scholarly attention. International research has mainly focused on telemedicine, digital home care, and home-based primary care, with particular emphasis on service accessibility, continuity of care, implementation barriers, and workforce readiness (5–8). These studies suggest that digital technologies can support the extension of nursing and care services from institutional settings to home and community contexts, especially in aging societies, while also highlighting persistent challenges related to technology adoption, professional capacity, and implementation sustainability. In contrast, Chinese studies have paid greater attention to the institutional evolution, regulatory arrangements, and policy expansion of internet-based healthcare. Existing research has examined the development trajectory of digital healthcare policies in China from the perspectives of legislative evolution, governance transformation, and institutional integration (9–11). Recent policy text analyses further indicate that Chinese internet-based healthcare policies are mainly oriented toward regulatory governance, service standardization, older adult care, and safety assurance, reflecting the strong policy-driven nature of this field (12). At the same time, studies using policy instrument analysis and quantitative text methods have shown that the policy mix of internet-based healthcare is generally characterized by the predominance of environment-oriented instruments and the insufficient coordination of supply- and demand-side instruments, which may weaken policy coherence and implementation sustainability (13). The PMC index model has also been increasingly applied in health policy research to evaluate the internal consistency and structural balance of policy design (14, 15). Overall, although domestic and international studies have generated important insights, their analytical emphases differ: international research is more concerned with service models, user outcomes, and implementation conditions, whereas domestic research focuses more on policy evolution, governance arrangements, and regulatory frameworks. Nevertheless, both strands of literature have rarely examined how policy themes, policy instrument configurations, and policy design consistency interact across governance levels within a unified analytical framework. As a result, important questions regarding multi-level institutional differentiation and structural coupling remain insufficiently addressed.

Amid the global acceleration of population aging and the rapid penetration of digital health technologies, the integration of internet technology with nursing services has transcended the realm of technological innovation to become a significant policy instrument for driving the structural transformation of long-term care systems. In many countries, efforts to extend nursing services from hospital-centered models toward community- and home-based settings are frequently confronted with institutional tensions between the logic of medical regulation and the logic of older adult care delivery. On one hand, there is a need to ensure medical safety and risk control; on the other, the accessibility and continuity of home-based care must be addressed. Consequently, the institutional integration of digital home-based nursing services has emerged as a critical issue in both digital health governance and long-term care reform. However, despite these important contributions, the theoretical contribution of existing research remains insufficiently specified. Current studies have mainly examined internet-based healthcare policies through relatively separate lenses, including institutional evolution, policy instrument allocation, and implementation conditions, but have rarely explained how these dimensions interact to shape policy structure, governance logic, and design consistency within a unified analytical perspective. This limitation is particularly evident in the field of “Internet Plus Nursing Services,” where policy development is shaped simultaneously by medical regulation, long-term care needs, and multi-level governance arrangements. As a result, the mechanisms through which thematic priorities, policy instrument configurations, and policy design quality jointly reflect structural differentiation across governance levels remain insufficiently understood.

Against this background, the present study offers novelty at three closely related levels. It integrates policy theme analysis, policy instrument analysis, and PMC-based evaluation into a three-dimensional analytical framework, moving beyond the fragmented use of single-method approaches in previous studies, and extends the empirical focus to China's “Internet Plus Nursing Services” policy domain, which is a distinctive setting for examining digital health governance under conditions of rapid institutional expansion, hierarchical coordination, and dual institutional embeddedness. More importantly, rather than treating the three dimensions as parallel analytical layers, the framework adopts a progressive structure in which policy themes capture issue attention and governance priorities, policy instruments reveal the institutional means through which these priorities are translated into action, and PMC-based evaluation assesses whether such priority-instrument arrangements remain structurally coherent and balanced at the policy design level. This integrated perspective thus advances understanding of how digital health policies evolve, differentiate, and coordinate across governance levels.

Accordingly, this study systematically examines the structural characteristics and design quality of “Internet Plus Nursing Services” policies from a multi-level governance perspective. It addresses three questions: what differences exist between national and local policies in terms of thematic focus and policy instrument configuration; how these differences are reflected in policy design consistency; and how dual institutional embeddedness shapes policy integration and coordination. In doing so, the study aims not only to compare policy arrangements across levels of government, but also to clarify the theoretical value of integrating policy instrument analysis with PMC-based evaluation in the study of digital health policy.

## 2 Materials and methods

### 2.1 Policy document selection and sample description

The policy system for “Internet Plus Nursing Services” is not constructed by a single level but constitutes a multi-level governance framework formed by overarching national policies and implementing local policies. National policies focus on establishing the institutional framework and fundamental norms, while local policies assume the function of specific implementation and operational refinement; the two are functionally distinct yet interdependent. This study integrates multi-source data, including the PKULAW database and the official websites of relevant government departments, such as the State Council, the National Health Commission, and various provincial and municipal Health Commissions. A systematic search was conducted using keywords including “Internet Plus Nursing Services”, “home-based nursing” and “continuity of care”. Policy documents were included if they met the following criteria: (1) officially issued by national or local government authorities; (2) directly related to “Internet Plus Nursing Services” or explicitly addressing home-based or internet-enabled nursing care; and (3) belonging to formal policy document types, such as policy notices, implementation plans, guiding opinions, and regulatory or normative documents. Documents that were irrelevant, duplicated, or lacking substantive policy content were excluded.

**Figure 1:**
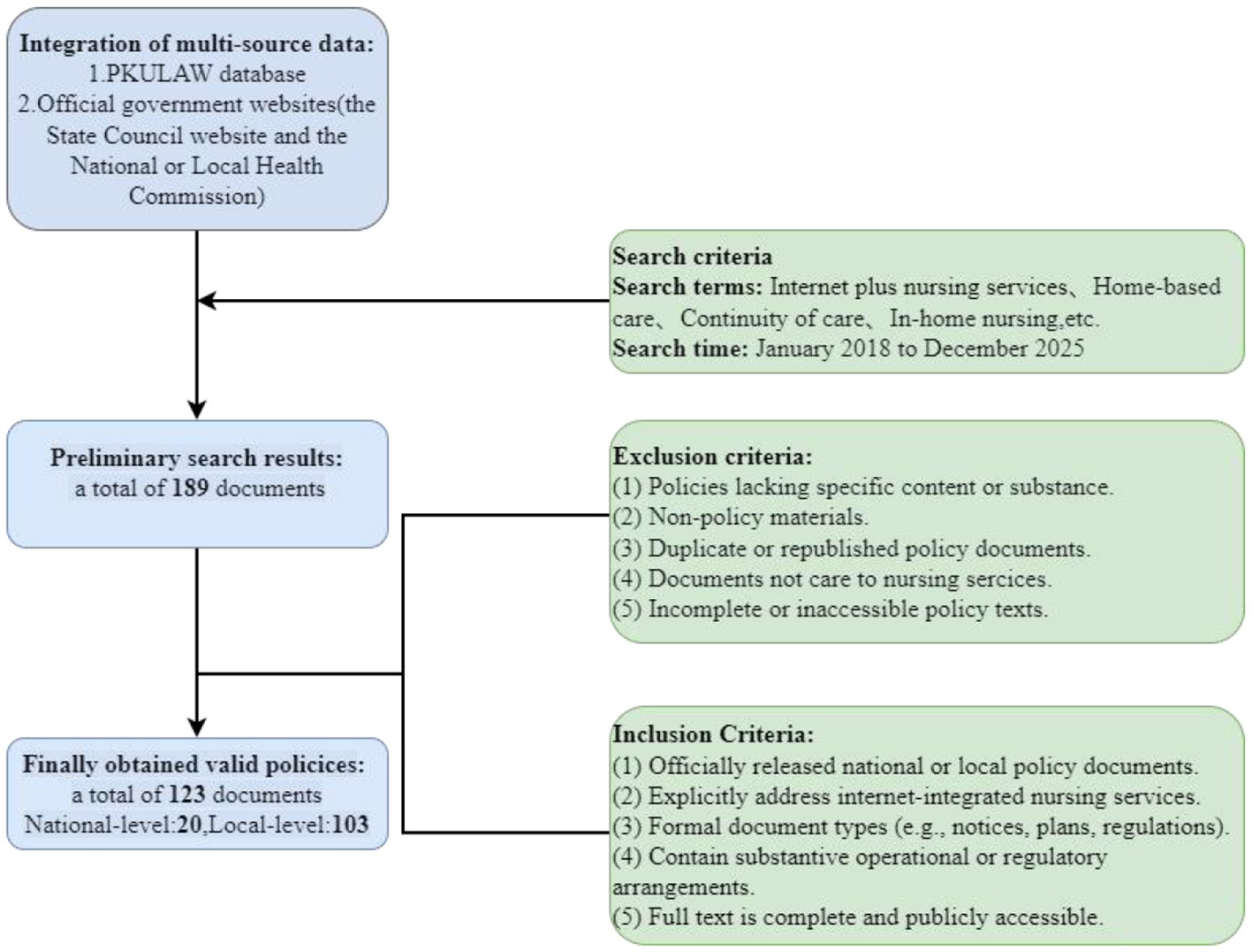
Data collection process for policy documents.

Given the limited policy texts in this field prior to 2018, the search period was confined to January 1, 2018, to December 31, 2025. The policy screening process followed predefined procedures, including identification, screening, eligibility assessment, and final inclusion, as illustrated in Figure 1. A total of 123 policy documents were ultimately included, comprising 20 at the national level and 103 at the local level. Among the local policies, documents were further distributed across different administrative levels, including provincial and municipal levels (with limited representation at lower administrative levels), reflecting the hierarchical structure of policy implementation. All policies have been numbered chronologically by their release date, and the complete list is provided in Supplementary material 1.

### 2.2 Overall analytical framework

**Figure 2:**
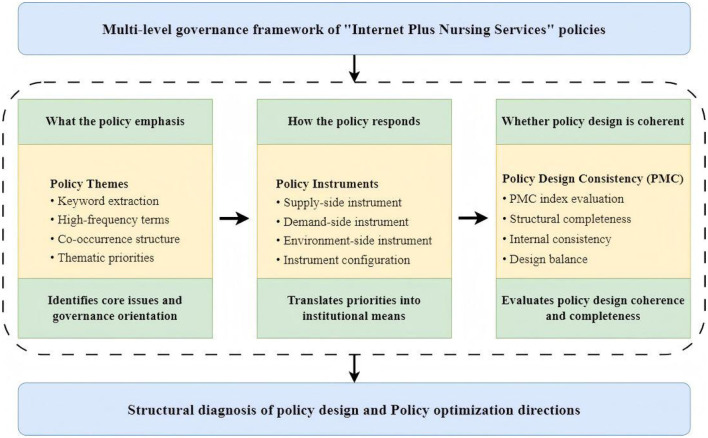
Three-dimensional analytical framework: policy keywords, instruments, and evaluation.

In this framework, the three dimensions are conceptually connected rather than mechanically juxtaposed. Policy theme analysis serves as the entry point for identifying substantive issues, governance priorities, and thematic orientations embedded in policy texts. Policy instrument analysis then examines how these priorities are translated into concrete institutional means, revealing the intervention logic and governance pathways through which policy objectives are operationalized. On this basis, PMC based evaluation provides a higher level assessment of whether the identified themes and instrument configurations are internally coherent, sufficiently balanced, and structurally complete. In other words, the three dimensions correspond to a progressive analytical logic of “what the policy emphasizes - how the policy responds - whether the policy is coherently designed.” Figure 2 has been revised accordingly to illustrate these inherent interconnections. To ensure the scientific rigor and validity of the assessment, this study contextualizes the policy paradigm within China's “Internet Plus Nursing Services” policy landscape. The policy analysis is deconstructed into key elements, including policy problems, policy goals, and policy instruments, and a three dimensional analytical framework of “Policy Keyword—Policy Instrument—Policy Evaluation” is constructed (Figure 2).

The keywords condensed from policy texts effectively reflect the core issues and focal points of the policies, and analyzing them helps grasp the overall orientation and central content of the policies (16). Therefore, this study first adopts the perspective of policy theme keywords, employing bibliometric methods to conduct word frequency analysis and semantic network analysis on the policy texts, so as to identify the fundamental characteristics and key areas of the policy system. Subsequently, given that policy instruments are the specific institutional means to achieve policy goals and reflect the modes and pathways of government intervention (17), this paper proceeds from the perspective of policy instruments. Utilizing content analysis, it systematically examines the configuration structure of supply oriented, demand oriented, and environment oriented policy instruments within the Internet Plus Nursing Services policies, aiming to reveal the intrinsic features of policy design. The resulting coding framework also informed the construction of the PMC variable system, particularly the operationalization of instrument related dimensions and associated governance functions, thereby linking the structural analysis of policy tools with the subsequent evaluation of policy design consistency. Furthermore, as policy evaluation constitutes a critical link in the formulation and implementation of public policy, its core lies in the systematic assessment of policy design quality (18). To this end, this study constructs a Policy Modeling Consistency (PMC) index model, conducting a comprehensive evaluation of Internet Plus Nursing Services policies from a multi dimensional structural perspective. This approach aims to identify structural deficiencies in policy design and provide references for subsequent policy optimization.

### 2.3 Policy theme identification and structural analysis

#### 2.3.1 Extraction and operationalization of policy themes

To identify the core themes and their associative structures within the policy texts on “Internet Plus Nursing Services,” this study employed Python-based natural language processing techniques to extract keywords and construct a keyword co-occurrence network for analysis. First, the policy texts underwent processing, including text cleaning and the removal of irrelevant characters. Subsequently, the JIEBA segmentation tool was utilized for word segmentation, and the results were subjected to manual verification and merging of terms in conjunction with the policy context to ensure the semantic integrity of professional terminology. Based on this, the TF-IDF method was applied to calculate word weights. By integrating term frequency and inverse document frequency, discriminative keywords were selected. High-frequency words were respectively chosen at the national and local levels to serve as nodes for the co-occurrence analysis.

Based on the aforementioned keywords, a co-occurrence matrix was constructed using policy sentences as the unit of analysis: if two words appeared in the same sentence, it was recorded as one co-occurrence, and their association strength was reflected through the cumulative frequency. Subsequently, the matrix was transformed into network data, and visualization and structural analysis were performed using Gephi software. The edge weights in the network corresponded to the co-occurrence frequencies, thereby differentiating the degree of association between various keywords.

#### 2.3.2 Coding and analysis of policy instruments

To systematically analyze the configuration and structural characteristics of policy instruments within the “Internet Plus Nursing Services” policies, this study employed MAXQDA software. Based on the policy instrument classification framework of Rothwell and Zegveld, a coding system was constructed, encompassing three main categories: supply oriented instruments, demand oriented instruments, and environment oriented instruments, together with several subcategories. Multiple rounds of revisions were undertaken to ensure its applicability. Individual policy clauses were used as the basic unit of analysis, and a total of 4,124 clause level units were identified from the policy corpus. Because one clause could simultaneously contain more than one substantively distinct policy instrument, multi code assignment was permitted where appropriate. As a result, the full coding process generated 9,336 coded references, which were used for the frequency based distributional analysis presented in Tables 1, 2. To improve methodological transparency, approximately 10.0% of the clause level units, corresponding to 412 units, were randomly selected for independent pilot coding by two trained researchers using the same coding manual. This pilot subsample generated 934 coding decisions. Inter coder reliability was assessed using Cohen's kappa coefficient. The kappa value was 0.842 for the three first level categories and 0.791 for the subcategory level coding, indicating substantial agreement. After the pilot coding round, disagreements were reviewed through discussion and the coding rules were further refined, particularly for clauses involving overlapping policy functions. During the formal coding process, all policy texts were imported into MAXQDA and coded clause by clause according to semantic meaning. When the content involved specific policy instruments or institutional arrangements, the corresponding code was assigned, and its location and frequency were recorded. Ambiguous cases were resolved through discussion and consensus. Finally, based on the coding results and operational definitions provided in Supplementary material 2, the distribution characteristics and temporal changes of policy instruments at the national and local levels were statistically analyzed. In addition, the coding framework provided an empirical basis for the operationalization of the PMC indicator system by informing the specification of policy instrument related variables used in the subsequent consistency evaluation.

**Table 1:** Distribution of policy instruments in national and local Internet plus nursing service policies.

Policy instrument category	Specific policy instruments	National level (*n*, %)	Local level (*n*, %)
Supply-side	Human resource development	121 (6.45%)	558 (7.48%)
	Health education and promotion	36 (1.92%)	265 (3.55%)
	Intelligent technology support	77 (4.11%)	1,036 (13.89%)
	Service capacity building	97 (5.17%)	594 (7.96%)
Demand-side	Medical insurance payment	183 (9.76%)	370 (4.96%)
	Pricing and fee regulation	113 (6.03%)	396 (5.31%)
	Commercial insurance participation	64 (3.41%)	294 (3.94%)
	Demand guidance mechanisms	88 (4.69%)	586 (7.85%)
Environment-side	Standards and specifications	349 (18.61%)	1,085 (14.54%)
	Supervision and regulation	333 (17.76%)	777 (10.41%)
	Institutional coordination	223 (11.89%)	299 (4.01%)
	Pilot programs and implementation mechanisms	201 (10.72%)	1,201 (16.10%)
Total coded units	–	1,875 (100.00%)	7,461 (100.00%)

**Table 2:** Stage-specific distribution of policy instruments at the national and local levels.

Policy instrument category	Specific policy instruments	Exploration (2018–2019)		Expansion (2020–2022)		Development (2023–2025)	
		National, *n* (%)	Local, *n* (%)	National, *n* (%)	Local, *n* (%)	National, *n* (%)	Local, *n* (%)
Supply-side	Human resource development	23 (7.31%)	144 (7.67%)	39 (6.26%)	234 (7.07%)	59 (6.28%)	180 (7.92%)
	Health education and promotion	7 (2.24%)	43 (2.29%)	13 (2.09%)	124 (3.75%)	16 (1.70%)	98 (4.31%)
	Intelligent technology support	18 (5.77%)	279 (14.86%)	27 (4.33%)	496 (14.98%)	32 (3.40%)	261 (11.48%)
	Service capacity building	19 (6.09%)	253 (13.48%)	34 (5.46%)	161 (4.86%)	44 (4.68%)	180 (7.92%)
Demand-side	Medical insurance payment	38 (12.18%)	70 (3.73%)	65 (10.43%)	167 (5.04%)	80 (8.51%)	133 (5.85%)
	Pricing and fee regulation	29 (9.29%)	45 (2.40%)	42 (6.74%)	167 (5.04%)	42 (4.47%)	184 (8.10%)
	Commercial insurance participation	11 (3.53%)	44 (2.34%)	21 (3.37%)	178 (5.38%)	32 (3.40%)	72 (3.17%)
	Demand guidance mechanisms	13 (4.17%)	68 (3.62%)	29 (4.65%)	263 (7.94%)	46 (4.89%)	255 (11.22%)
Environment-side	Standards and specifications	44 (14.10%)	175 (9.32%)	96 (15.41%)	535 (16.16%)	209 (22.23%)	375 (16.50%)
	Supervision and regulation	42 (13.46%)	169 (9.00%)	87 (13.96%)	390 (11.78%)	204 (21.70%)	218 (9.59%)
	Institutional coordination	38 (12.18%)	130 (6.93%)	82 (13.16%)	98(2.96%)	103 (10.96%)	71 (3.12%)
	Pilot programs and implementation mechanisms	40 (12.82%)	457 (24.35%)	88 (14.13%)	498 (15.04%)	73 (7.77%)	246 (10.82%)
Total coded references	–	312 (100.00%)	1,877 (100.00%)	623 (100.00%)	3,311 (100.00%)	940 (100.00%)	2,273 (100.00%)

#### 2.3.3 Evaluation of policy design consistency

To assess the structural completeness and internal consistency of “Internet Plus Nursing Services” policies, this study employed the Policy Modeling Consistency (PMC) index model. PMC scoring was based on the full texts of formally issued and currently effective policy documents selected from the national and local policy corpus. National-level samples were primarily obtained from the State Council and relevant authorities under the National Health Commission, whereas local-level samples were drawn from provincial and sub-provincial governments and their health administrative departments. To ensure comparability and interpretability, representative policy documents with clear structure, substantive content, and sufficient information for multidimensional evaluation were purposively selected. Accordingly, 12 national and 12 local policy documents were included, and the full sample list is presented in Tables S10, S11 in Supplementary material 3.

The PMC variable system was developed with reference to Ruiz Estrada's framework and adapted to the policy context of “Internet Plus Nursing Services.” The final framework comprised nine first-level variables (X1–X9) and 35 second-level variables, covering policy nature, policy instruments, policy effectiveness, policy scope, policy objectives, policy goals, policy function, incentives and constraints, and policy evaluation. The structure of the PMC indicator system and the corresponding evaluation criteria are presented in Table 3. Based on the preliminary policy instrument coding results, the PMC indicator system was further operationalized for application to the selected policy documents. Specifically, the coding framework informed the specification of instrument-related and governance-related PMC dimensions, thereby establishing an analytical linkage between the structural analysis of policy tools and the subsequent consistency evaluation.

**Table 3:** First-level and second-level variables of the PMC index system.

First-level variables	Secondary-level variables	Evaluation standards
X1 Policy nature	X1.1 Predictive	Judge whether the policy document explicitly demonstrates identifiable policy attributes, including predictive, advisory, descriptive, guiding, or regulatory characteristics. If yes, a score of one is assigned, otherwise zero
	X1.2 Advisory	
	X1.3 Descriptive	
	X1.4 Guiding	
	X1.5 Regulatory	
X2 Policy instruments	X2.1 Supply-side	Judge whether the policy clearly adopts specific supply-side, demand-side, or environment-side instruments to intervene in the policy domain. If yes, a score of one is assigned, otherwise zero
	X2.2 Demand-side	
	X2.3 Environment-side	
X3 Policy effectiveness	X3.1 Temporary	Judge whether the policy impact is within 1 year, 1–5 years, 6–10 years, or more than 10 years. If yes, a score of one is assigned, otherwise zero
	X3.2 Short-term	
	X3.3 Medium-term	
	X3.4 Long-term	
X4 Policy scope	X4.1 Coverage	Judge whether the policy clearly defines its scope in terms of target population, service setting, coverage range, or applicability. If yes, a score of one is assigned, otherwise zero
	X4.2 Population	
	X4.3 Setting	
	X4.4 Scalability	
X5 Policy objectives	X5.1 Access	Judge whether the policy explicitly articulates objectives aimed at improving access, quality, efficiency, equity, or continuity of services. If yes, a score of one is assigned, otherwise zero
	X5.2 Quality	
	X5.3 Efficiency	
	X5.4 Equity	
	X5.5 Continuity	
X6 Policy goals	X6.1 Expansion	Judge whether the policy sets outcome-oriented goals indicating expected results, such as expansion, standardization, integration, sustainability, or system optimization. If yes, a score of one is assigned, otherwise zero
	X6.2 Standardization	
	X6.3 Integration	
	X6.4 Sustainability	
	X6.5 Qptimization	
X7 Policy function	X7.1 Regulatory constraints	Judge whether the policy performs identifiable functional roles within the governance system, including regulation, guidance, service optimization, innovation promotion, or technological support. If yes, a score of one is assigned, otherwise zero
	X7.2 Normative guidance	
	X7.3 Service potimization	
	X7.4 Innovation-driven	
	X7.5 Technology support	
X8 Incentives and constraints	X8.1 Safeguards	Judge whether the policy employs concrete incentive or constraint mechanisms, such as safeguards, monitoring arrangements, training measures, staffing provisions, implementation mechanisms, or funding support. If yes, a score of one is assigned, otherwise zero
	X8.2 Monitoring	
	X8.3 Training	
	X8.4 Staffing	
	X8.5 Implementation	
	X8.6 Funding	
X9 Policy evaluation	X9.1 Indicators	Judge whether the policy establishes explicit evaluation-related arrangements, including indicators, monitoring processes, feedback mechanisms, or adjustment procedures. If yes, a score of one is assigned, otherwise zero
	X9.2 Monitoring	
	X9.3 Feedback	
	X9.4 Adjustment	

All second-level variables were assigned equal weights and coded using a binary scoring approach. A score of 1 was assigned when the policy text explicitly and substantively addressed the corresponding indicator, and 0 otherwise. First-level scores were calculated by aggregating the relevant second-level indicators, on which the overall PMC index was subsequently derived. For interpretive purposes, PMC scores were further classified into four categories—perfect, excellent, qualified, and low—according to the criteria shown in Table S10 in Supplementary material 3. Statistical analyses were conducted in SPSS, and PMC surface plots were generated using MATLAB to visualize structural differences across policy samples.

## 3 Results

### 3.1 Descriptive characteristics of national and local policy documents

A total of 123 policy documents were included, comprising 20 national-level policies (16.26%) and 103 local-level policies (83.74%). This distribution reflects the multi-level structure of “Internet Plus Nursing Services” governance in China and provides the empirical basis for subsequent comparison between national and local policies. In addition to differences in document quantity, the two levels also differed markedly in textual scale: the mean word count of local policies was substantially higher than that of national policies (8,203 vs. 2,007 words), suggesting that local policies were more strongly oriented toward implementation detail and operational elaboration, whereas national policies mainly played a framework-setting role. In terms of issuing patterns, health administrative departments accounted for the largest share of policy issuers (72.36%), indicating their central role in shaping this policy domain. The relevant descriptive characteristics are presented in Table 4.

**Table 4:** Descriptive overview of national and local policy texts.

Category	Number of policies	Percentage (%)	Mean words count
Policy level			
National	20	16.26	2,007
Local	103	83.74	8,203
Policy issuing authority			
Health administration departments	89	72.36	9,909
Jointly issued policies	34	27.64	301
Total	123	100	10,210

### 3.2 Hierarchiacal differences in policy themes and co-occurrence structures

#### 3.2.1 Salient policy themes across national and local policies

As shown in Table 5, the comparative keyword results revealed clear hierarchical differences in policy themes between the national and local levels. At the national level, high-frequency terms were more strongly concentrated around governance- and system-related concepts, such as Service (*n* = 929), Internet Plus Nursing Service (*n* = 589), Care Seeking (*n* = 374), Healthcare (*n* = 273), and Health Insurance (*n* = 218), indicating that national policies primarily framed the development of “Internet Plus Nursing Services” within a broader institutional and regulatory architecture. By contrast, local policies placed greater emphasis on service recipients and delivery settings, as reflected by the prominence of older adult care (*n* = 4,766), Older Adults (*n* = 2,860), Home-based Nursing (*n* = 1,970), Limited Mobility (*n* = 1,923), and Primary Level (*n* = 1,593). This contrast suggests that national policies were more strongly oriented toward system governance and institutional coordination, whereas local policies focused more directly on target populations, community-based care scenarios, and practical service delivery arrangements.

**Table 5:** Comparative high-frequency policy themes at the national and local levels (top 15 keywords).

Rank	National-level keyword	Frequency	Local-level keyword	Frequency
1	Service	929	Older adult care	4,766
2	Internet plus nursing service	589	Older adults	2,860
3	Care seeking	374	Home-based nursing	1,970
4	Supply	273	Limited mobility	1,923
5	Healthcare	273	Primary level	1,593
6	Online	219	Internet plus nursing services	1,080
7	Health insurance	218	Community	1,051
8	Patients	206	Public health	1,029
9	Medical care	196	Services	852
10	Home-based care	191	Healthcare	789
11	Coordination	187	Patients	754
12	Basic	183	Assessment	737
13	Capacity	180	Regulation	737
14	Enrollment	177	Standards	666
15	Service	929	Safeguards	655

**Figure 3:**
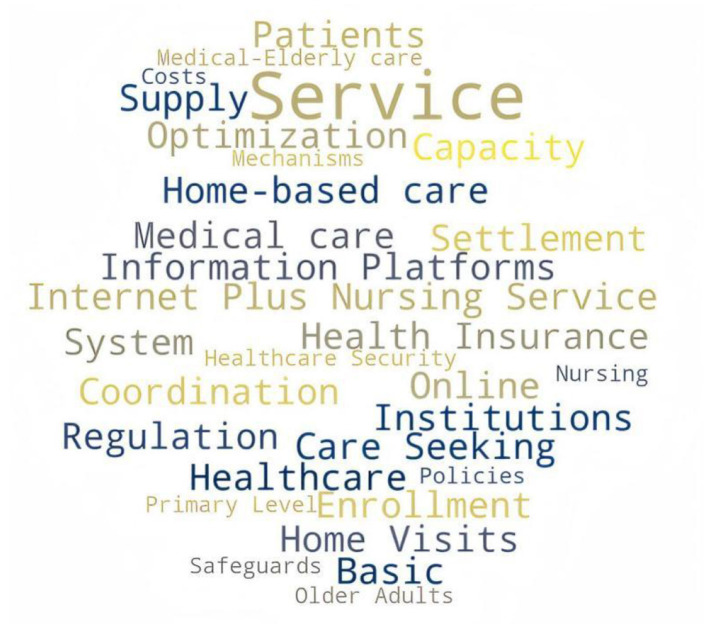
Word cloud of high-frequency policy themes in national Internet-based nursing service policies (selected).

**Figure 4:**
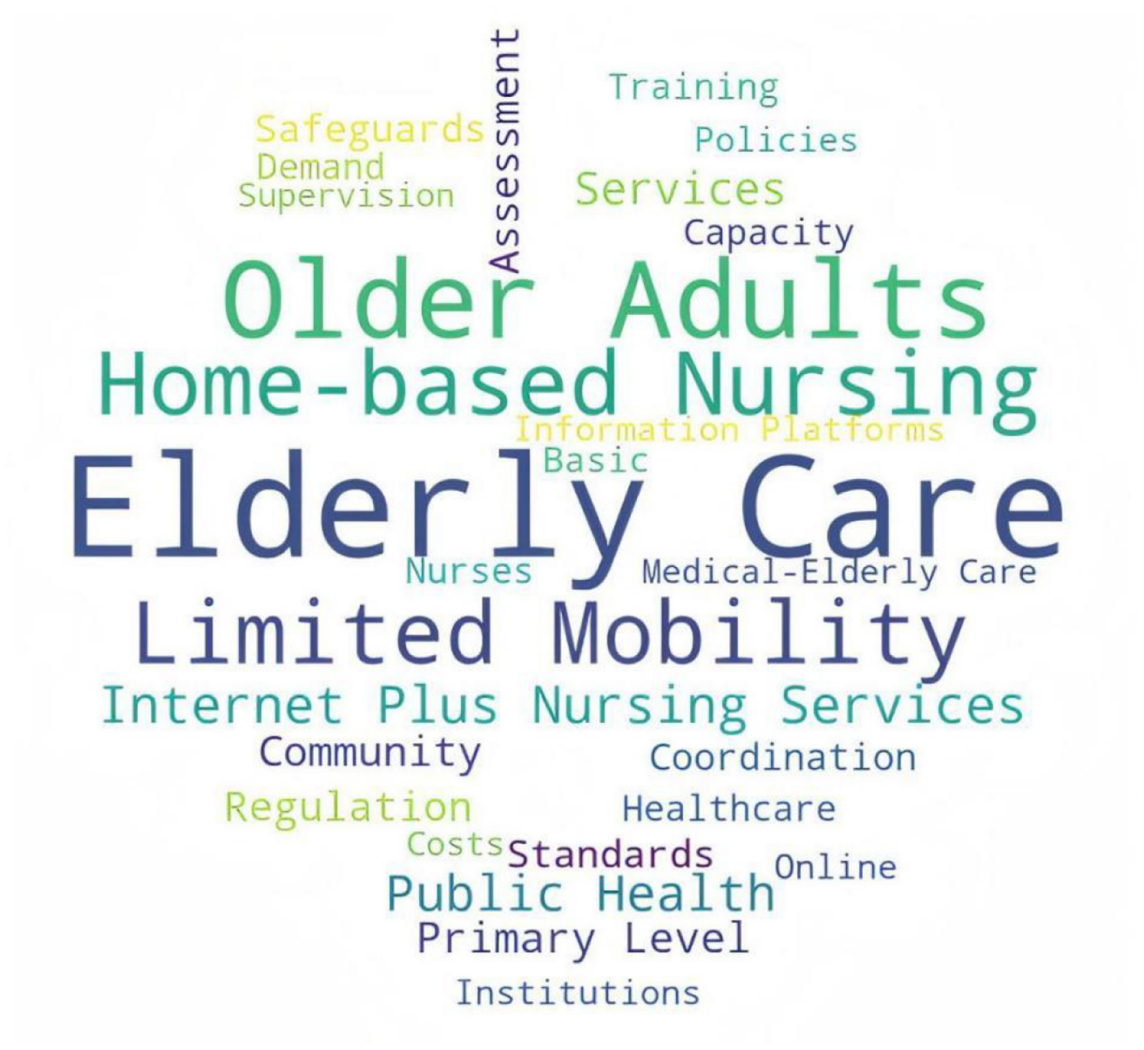
Word cloud of high-frequency policy themes in local Internet-based nursing service policies (selected).

Figures 3, 4 provide a visual complement to the comparative keyword results presented in Table 5. The national word cloud is more concentrated around governance- and system-related themes, whereas the local word cloud places greater visual emphasis on service recipients, care settings, and implementation scenarios. This visual contrast further supports the cross-level difference identified in Table 5, namely that national policies were more strongly oriented toward institutional coordination and regulatory design, while local policies were more closely aligned with practical service delivery and community-based implementation.

#### 3.2.2 Cross-level patterns in policy theme co-occurrence

**Figure 5:**
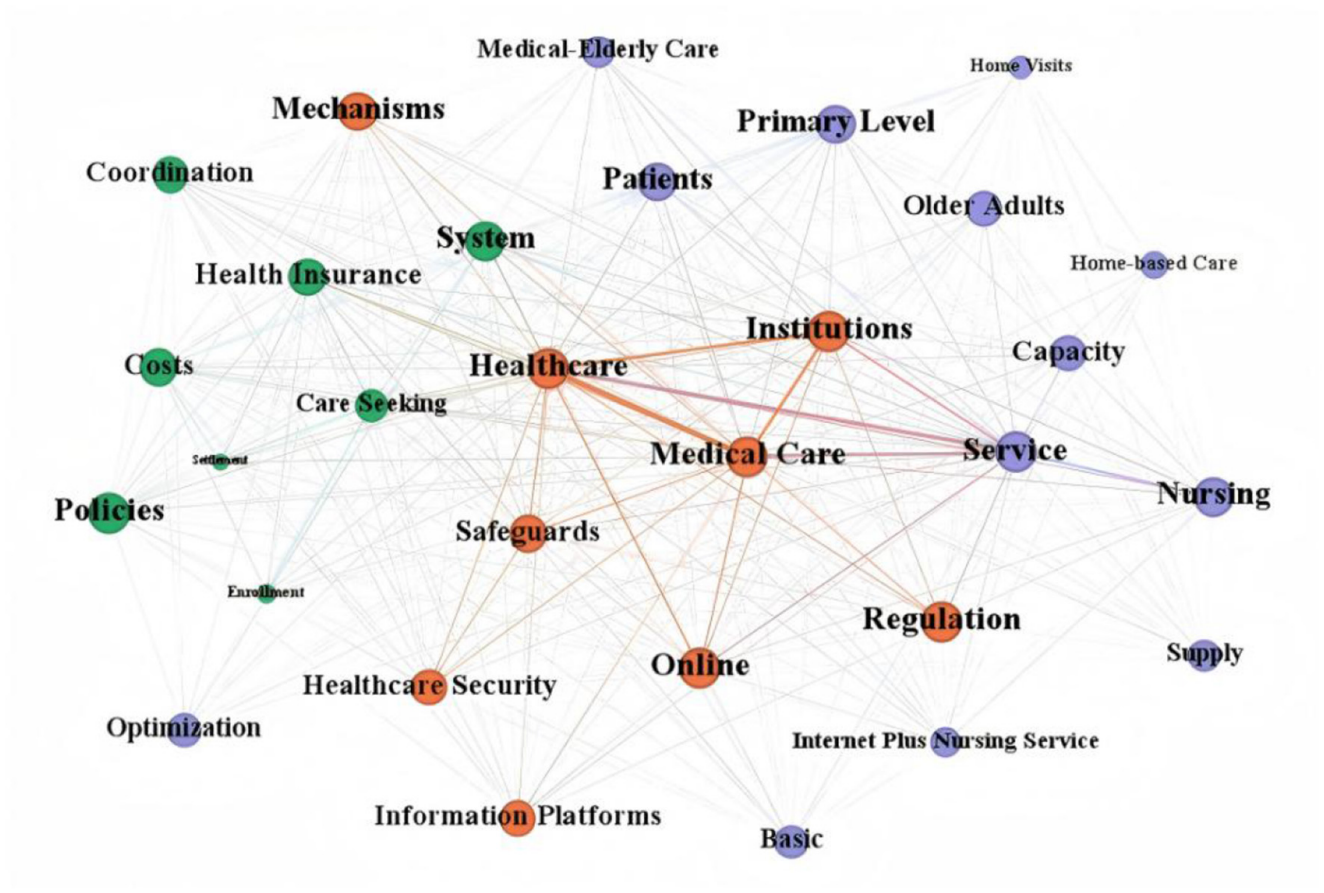
National-level co-occurrence network of internet-based nursing service policy keyword.

**Figure 6:**
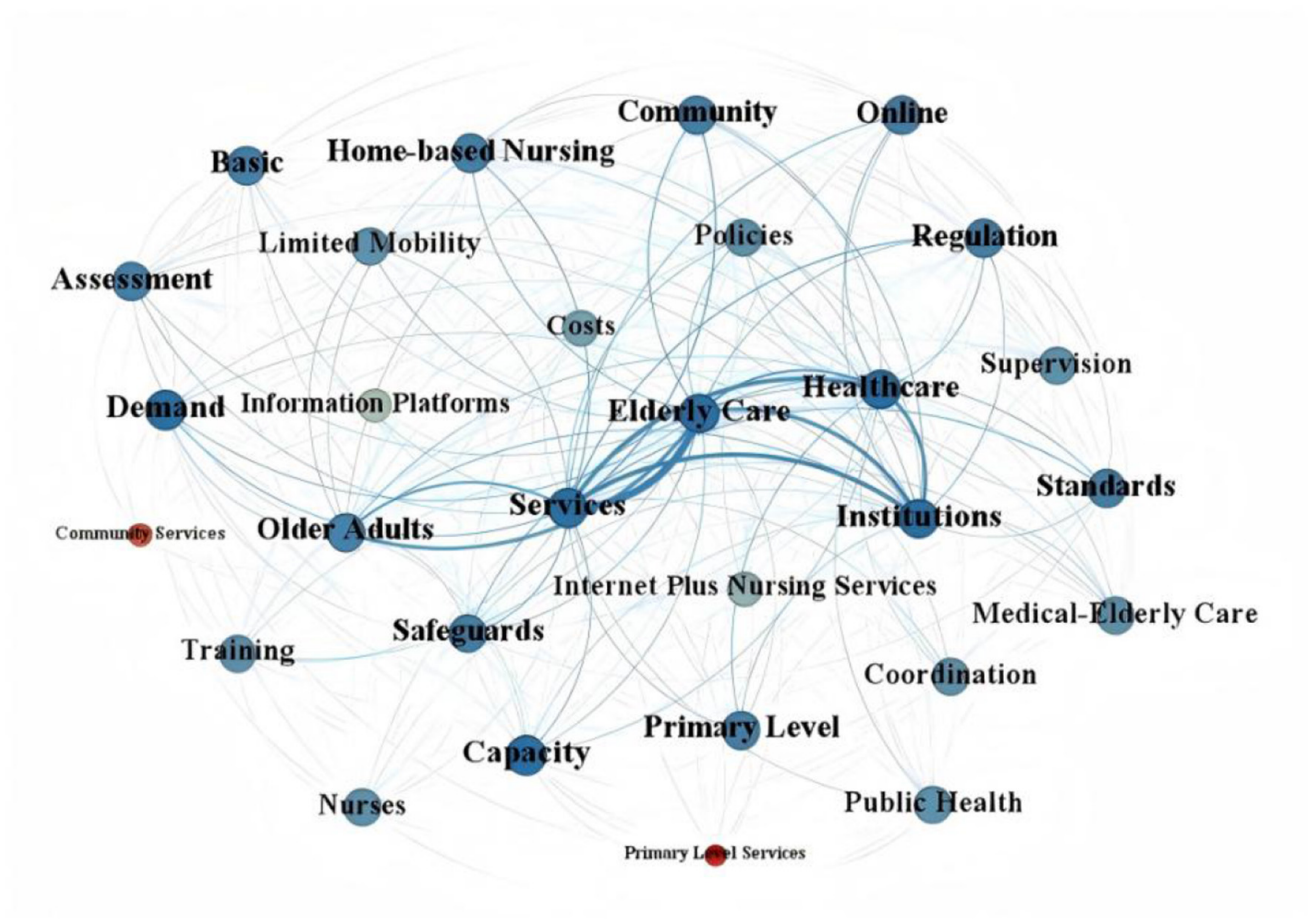
Local-level co-occurrence network of internet-based nursing service policy keyword.

The co-occurrence networks shown in Figures 5, 6 further revealed clear hierarchical differences in the internal thematic structures of national and local policies. Overall, the national network exhibited a more centralized and governance-oriented structure, whereas the local network showed a more decentralized and practice-oriented pattern. This difference suggests that, although both levels addressed the development of “Internet Plus Nursing Services,” they differed substantially in the way policy themes were internally organized and connected.

At the national level, the network was organized around a relatively concentrated core composed of terms such as Healthcare, Medical Care, Service, and Nursing, with governance-related terms including Regulation, Safeguards, and Healthcare Security maintaining close connections with the core nodes. This pattern indicates that national policies primarily embedded Internet-based nursing services within a broader framework of institutional governance, regulatory coordination, and system safety.

By contrast, the local network displayed a more dispersed and implementation-centered configuration. Its core structure was formed around terms such as older adult care, Older Adults, Home-based Nursing, and Limited Mobility, which were more closely linked to service delivery and capacity-related themes such as Community, Primary Level, Training, and Capacity. Taken together, these findings suggest that national policies were more strongly oriented toward institutional integration and regulatory design, whereas local policies focused more directly on service implementation, target populations, and grassroots care delivery.

### 3.3 Policy instrument configurations and stage-specific shifts across governance levels

As indicated in Table 1, environment-oriented instruments dominated policy design at both governance levels, confirming that “Internet Plus Nursing Services” policies were primarily advanced through regulatory and governance arrangements. However, their internal composition differed substantially. National policies were more strongly concentrated in standards and specifications, supervision and regulation, and institutional coordination, whereas local policies relied more heavily on pilot programs and implementation mechanisms and intelligent technology support. This contrast suggests that national policies were more strongly oriented toward constructing the regulatory architecture of the policy domain, while local policies placed greater weight on implementation support and service operationalization.

Stage-specific analysis further revealed different trajectories of policy adjustment across governance levels. In the exploratory stage, local policies depended more heavily on pilot-oriented implementation tools, whereas national policies already showed a stronger concentration in regulatory and coordination-related instruments. During the expansion stage, both levels moved toward greater standardization, but this shift was expressed more through regulatory consolidation nationally and through the simultaneous strengthening of standards and implementation support locally. By the development stage, national policies became increasingly concentrated in standards and supervision, whereas local policies showed growing prominence of demand guidance and pricing-related instruments, indicating a gradual shift from pilot mobilization toward more service-responsive governance.

### 3.4 PMC-based assessment of policy design consistency across governance levels

#### 3.4.1 Cross-level differences and structural imbalances in PMC index results

As shown in Table 6, clear cross-level differences were observed in PMC performance between national and local policies. National policies achieved a higher overall mean PMC index than local policies (7.98 vs. 7.31), indicating a comparatively stronger level of policy design consistency. At the dimensional level, both policy groups performed relatively well in several core governance dimensions, but notable structural imbalances remained. National policies showed particularly high mean scores in X1, X2, X6, and X7 (0.97–0.98), whereas lower scores were observed in X3 Policy coverage (0.56) and X9 Policy evaluation (0.75). A similar but more pronounced pattern was found at the local level, where X7 and X4 remained relatively strong (0.97 and 0.96), but X3 declined to 0.27 and X9 to 0.73. These cross-level differences were closely related to the thematic emphases and instrument configurations identified in the preceding analyses, but their significance extends beyond isolated low-scoring dimensions. Rather, the PMC results indicate a broader pattern of uneven policy maturation across governance levels. National policies, characterized by a stronger focus on standards, supervision, and institutional coordination, performed more strongly in dimensions associated with framework construction and governance completeness. By contrast, local policies, while maintaining relatively strong performance in implementation-oriented dimensions, showed weaker results in dimensions more closely related to long-term policy effectiveness, goal integration, and evaluation arrangements. This suggests that local policy expansion was driven more strongly by operational problem-solving and service delivery needs than by the simultaneous consolidation of design completeness and feedback-oriented governance.

**Table 6:** Mean PMC scores by demension at the national and local levels.

PMC dimension	National mean	Local mean	Difference
X1 Policy nature	0.97	0.80	0.17
X2 Policy instruments	0.97	0.95	0.02
X3 Policy effectiveness	0.56	0.27	0.29
X4 Policyscope	0.96	0.96	0.00
X5 Policy objectives	0.92	0.86	0.06
X6 Policy goals	0.98	0.86	0.12
X7 Policy functions	0.98	0.97	0.01
X8 Incentives and constraints	0.90	0.92	0.02
X9 Policy evaluation	0.75	0.73	0.02
Overall PMC index	7.98	7.31	0.67

Detailed policy-level results in Tables 7, 8 further illustrate that the cross-level gap identified in Table 6 was reflected not only in average PMC scores, but also in the distribution of higher- and lower-performing cases within each policy group. This pattern was also evident at the individual policy level. For example, the lowest-scoring national policy (N3) showed concurrent weaknesses in policy effectiveness, policy objectives, and policy evaluation, suggesting that lower PMC performance was associated with multidimensional structural deficiencies rather than with a single isolated shortcoming. This finding further indicates that weaker policies tended to be characterized by cumulative design gaps across several dimensions.

**Table 7:** PMC indices of selected national-level “Internet Plus Nursing Service” policies.

	X1	X2	X3	X4	X5	X6	X7	X8	X9	PMC index	Rating	Ranking
N1	1.00	1.00	0.75	1.00	0.80	1.00	1.00	1.00	0.50	8.05	Excellent	9
N2	1.00	1.00	1.00	1.00	1.00	1.00	1.00	1.00	0.75	8.75	Excellent	1
N3	0.60	0.67	0.25	0.75	0.40	0.80	0.80	0.67	0.25	5.19	Qualified	12
N4	1.00	1.00	0.75	1.00	1.00	1.00	1.00	1.00	0.75	8.50	Excellent	2
N5	1.00	1.00	0.25	0.75	0.80	1.00	1.00	0.67	0.50	6.72	Qualified	11
N6	1.00	1.00	0.75	1.00	1.00	1.00	1.00	1.00	0.75	8.50	Excellent	2
N7	1.00	1.00	0.50	1.00	1.00	1.00	1.00	0.67	0.75	7.92	Excellent	10
N8	1.00	1.00	0.50	1.00	1.00	1.00	1.00	0.83	1.00	8.33	Excellent	7
N9	1.00	1.00	0.50	1.00	1.00	1.00	1.00	1.00	1.00	8.50	Excellent	2
N10	1.00	1.00	0.50	1.00	1.00	1.00	1.00	1.00	0.75	8.25	Excellent	8
N11	1.00	1.00	0.50	1.00	1.00	1.00	1.00	1.00	1.00	8.50	Excellent	2
N12	1.00	1.00	0.50	1.00	1.00	1.00	1.00	1.00	1.00	8.50	Excellent	2

**Table 8:** PMC indices of selected local-level “Internet Plus Nursing Service” policies.

	X1	X2	X3	X4	X5	X6	X7	X8	X9	PMC index	Rating	Ranking
L1	0.80	1.00	0.00	0.75	0.60	0.80	1.00	0.83	0.50	6.28	Qualified	11
L2	0.80	1.00	0.25	1.00	0.80	1.00	1.00	1.00	0.75	7.60	Excellent	5
L3	0.80	1.00	0.25	1.00	0.80	0.80	1.00	0.83	0.75	7.23	Excellent	9
L4	0.80	1.00	0.75	0.75	1.00	0.75	1.00	1.00	1.00	8.05	Excellent	1
L5	0.80	1.00	0.25	1.00	0.80	0.80	1.00	1.00	0.75	7.40	Excellent	8
L6	0.80	0.67	0.25	1.00	1.00	1.00	1.00	1.00	1.00	7.72	Excellent	4
L7	0.80	0.67	0.25	1.00	0.60	0.80	0.80	0.50	0.25	5.67	Qualified	12
L8	0.80	1.00	0.25	1.00	1.00	1.00	1.00	1.00	0.75	7.80	Excellent	2
L9	0.80	1.00	0.25	1.00	1.00	0.80	0.80	0.83	0.50	6.98	Qualified	10
L10	0.80	1.00	0.25	1.00	0.75	1.00	1.00	1.00	1.00	7.80	Excellent	2
L11	0.80	1.00	0.25	1.00	1.00	0.80	1.00	1.00	0.75	7.60	Excellent	5
L12	0.80	1.00	0.25	1.00	1.00	0.80	1.00	1.00	0.75	7.60	Excellent	5

Within the national sample, PMC scores were concentrated largely in the upper range, with only a small number of policies falling into the qualified category. This distribution indicates that national policy design was generally stable in its internal consistency, even though some variation remained across individual documents. The observed score range suggests that higher-level policy design was not uniformly balanced in every case; rather, weaker policies tended to be marked by shortfalls in dimensions related to policy coverage and evaluation. In this sense, the national group combined relatively strong overall performance with limited but still meaningful internal heterogeneity.

The local sample displayed a less concentrated score distribution and a larger share of qualified cases, pointing to greater heterogeneity in policy design consistency. Although several local policies reached relatively strong PMC levels, lower-scoring cases were more common than in the national group, indicating that structural weaknesses were more frequently embedded in local policy design. This dispersion is consistent with the dimensional pattern reported above, particularly the weaker performance in coverage- and evaluation-related dimensions. Overall, the local group appeared less uniformly balanced across policy documents, despite the presence of several comparatively strong cases.

#### 3.4.2 Structural profiles of policy design consistency

Building on the preceding analyses, the PMC profiles further indicate that cross-level differences in policy design consistency were rooted in distinct policy structuring logics. The stronger national PMC performance corresponded to the national emphasis on institutional embedding, regulatory coordination, and standards-based governance, which was more readily translated into higher scores in governance-related dimensions. In contrast, the local emphasis on pilot implementation, intelligent technology support, and community-based service delivery strengthened operational responsiveness, but did not necessarily produce comparable completeness in policy scope, evaluation, and feedback arrangements.

**Figure 7:**
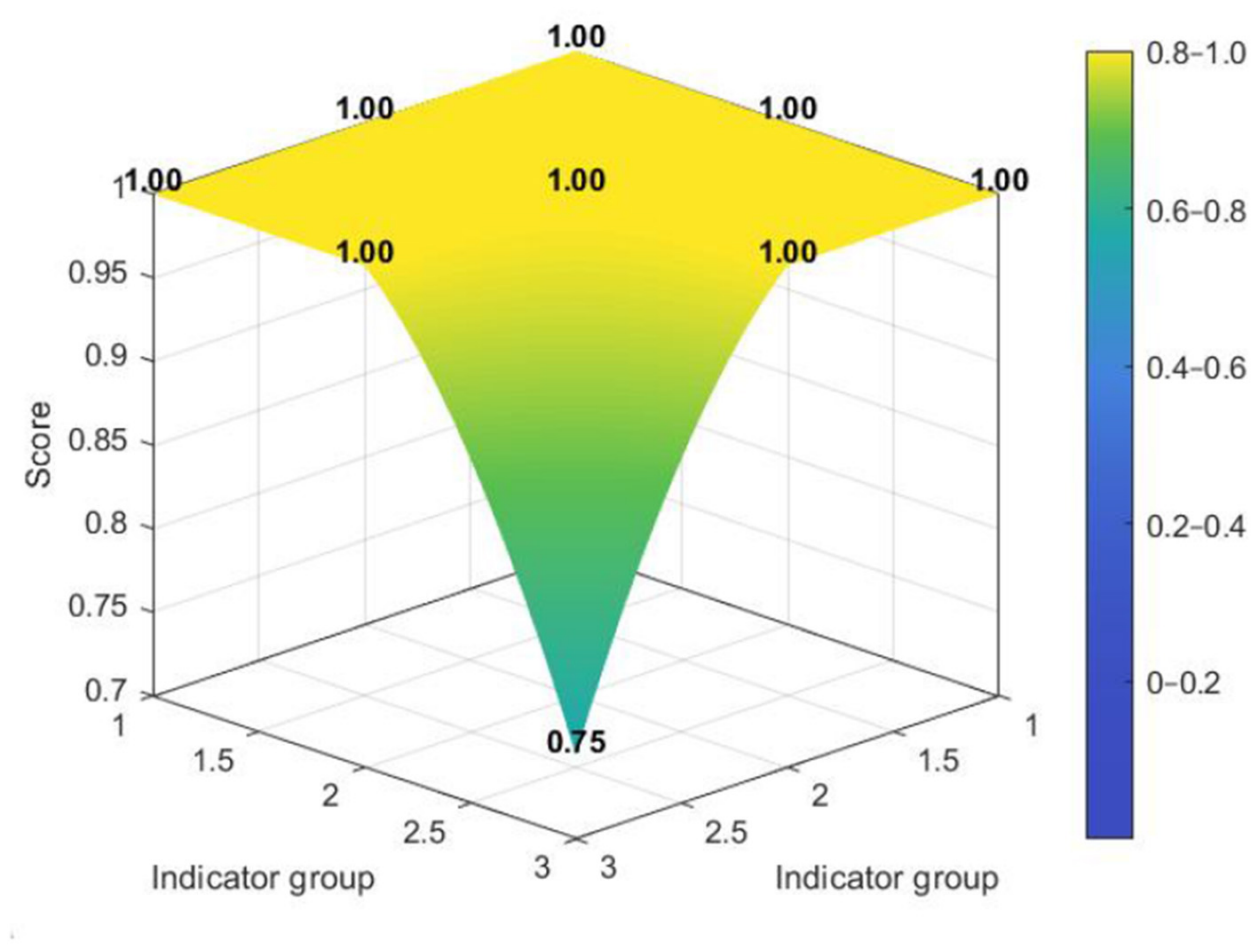
PMC surface of higher-scoring national policy N2.

**Figure 8:**
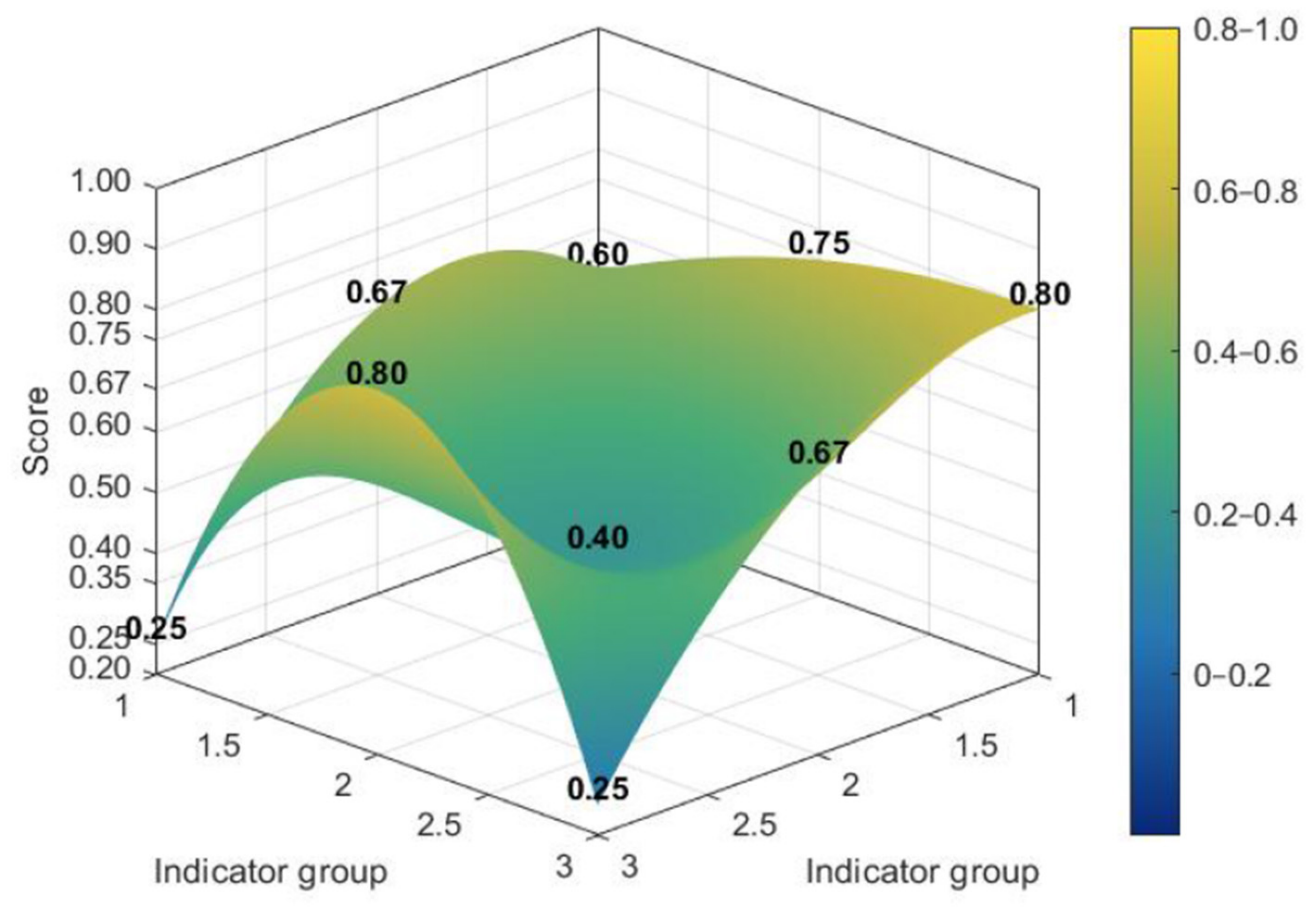
PMC surface of lower-scoring national policy N3.

**Figure 9:**
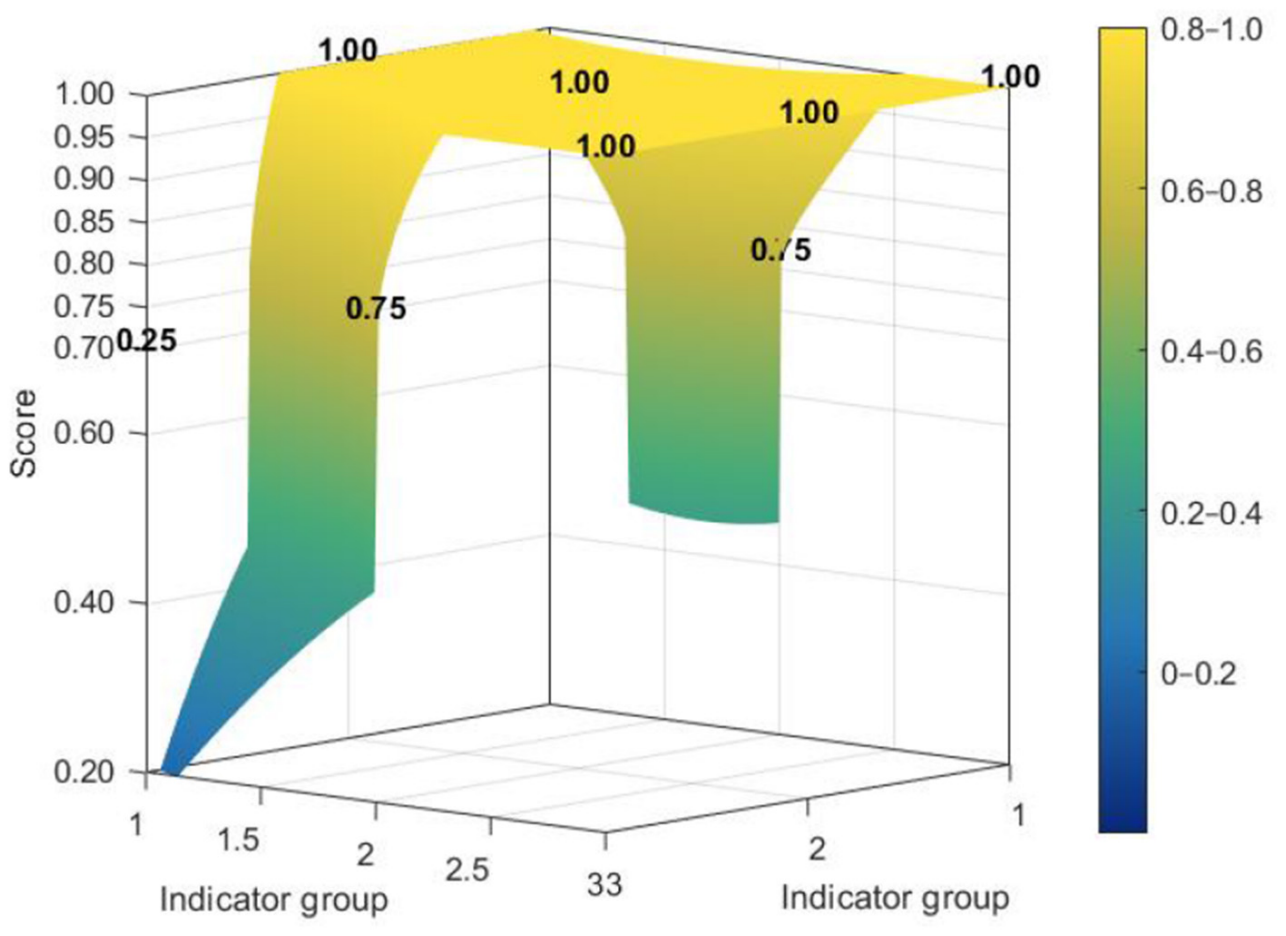
PMC surface of higher-scoring national policy L4.

**Figure 10:**
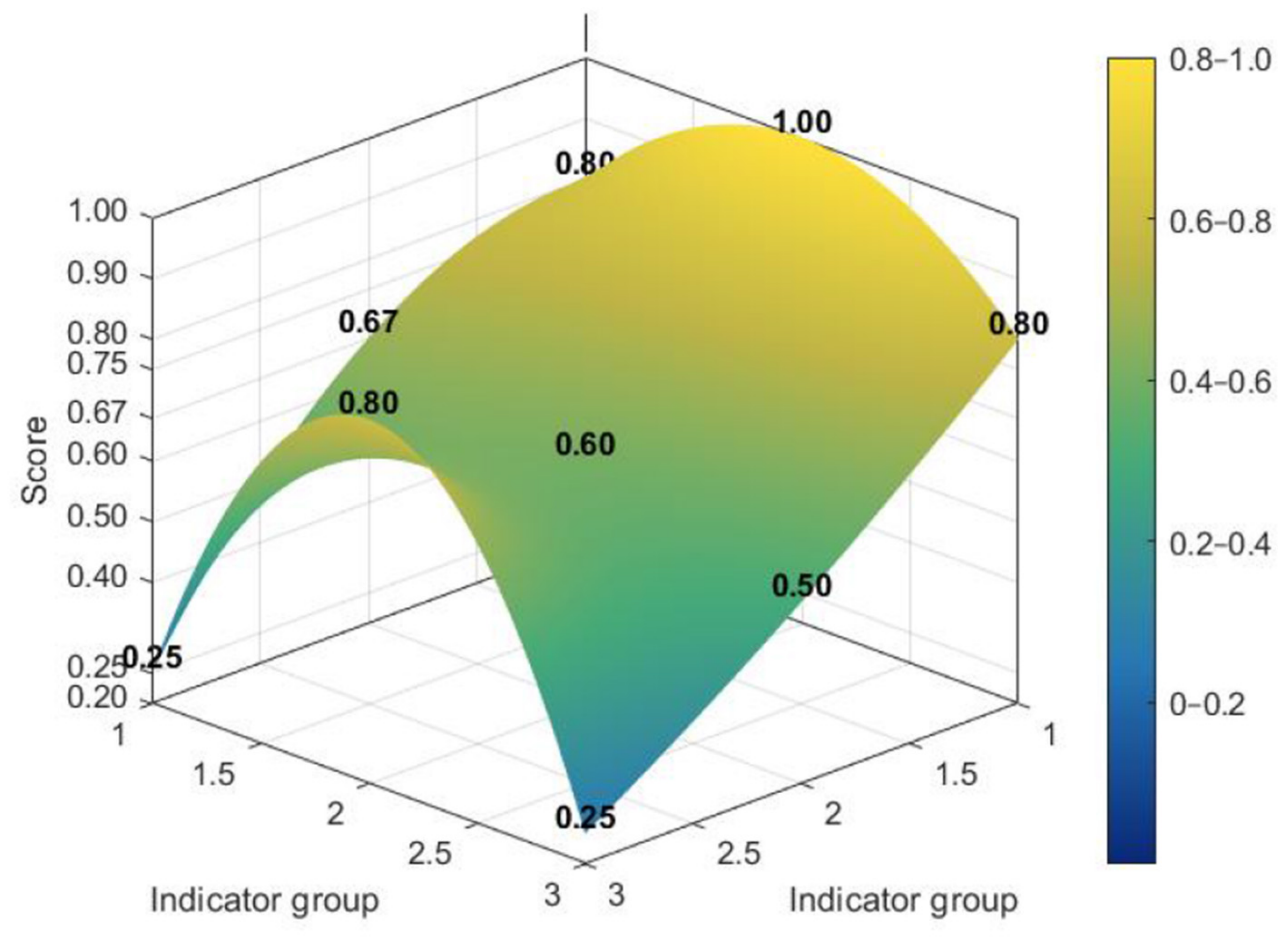
PMC surface of lower-scoring national policy L7.

Figures 7–10 make visible an additional layer of cross-level difference by showing how policy consistency was distributed within the dimensional structure of individual policies. Higher-scoring policies tended to display smoother and more even PMC surfaces, whereas lower-scoring policies were characterized by sharper depressions in selected dimensions. The distinction therefore lies not only in score level, but also in the extent to which different dimensions were coordinated within the policy design.

Among national policies, stronger cases exhibited a relatively integrated surface profile across core governance dimensions, while weaker cases showed more evident deficits in policy coverage and policy evaluation. This suggests that the national policy framework was generally robust in its regulatory orientation and institutional coordination, but could still lose coherence where scope definition and evaluative follow-up were insufficiently specified.

The contrast was more pronounced at the local level. Although some local policies achieved relatively strong structural performance, weaker cases showed deeper imbalances across dimensions, especially in areas associated with coverage, evaluation, and supporting arrangements. Read together with the preceding analyses, these profiles suggest that local policies were comparatively more effective in operationalizing implementation and service delivery than in maintaining a fully balanced design structure. A radar chart comparing the dimensional profiles of national and local PMC scores is provided in Supplementary material 3.

## 4 Discussion

### 4.1 Hierarchical differentiation of policy themes and divergence in governance focus: the case of internet plus nursing service

The systematic divergence in policy themes concerning “Internet Plus Nursing Services” between the national and local levels is an inevitable outcome of functional division under a multi-level governance framework (19, 20). Central policies are highly concentrated on institutional frameworks, payment regulations, and governance oversight, with the keyword network centering on the healthcare system as the core node. This reflects a governance orientation that anchors “Internet Plus Nursing Services” as an extension of the medical service system. In contrast, local policies show a significant tilt toward older adult care, home-based services, and community settings, with a more decentralized network structure, reflecting a practical preference for integrating these services into the spectrum of community home-based older adult care. This pattern clearly indicates that “Internet Plus Nursing Services” carries distinct functional orientations at different levels within the same policy domain: the central level emphasizes standardization, unification, and risk prevention, while the local level focuses on service implementation and contextual adaptation.

This cross-level differentiation was not merely a result of administrative division, but was deeply shaped by China's central–local governance structure, interdepartmental coordination arrangements, and pilot-based policy tradition. As shown in the results, national policies were more strongly concentrated in standards, supervision, and institutional coordination, whereas local policies placed greater emphasis on pilot implementation and technology support. This pattern reflects differentiated governance responsibilities across levels. At the national level, central authorities are more directly responsible for controlling medical risk, maintaining policy consistency, and defining regulatory boundaries in a policy domain involving home-based care, online platforms, patient safety, and data governance (21, 22). In addition, because this policy domain simultaneously involves health administration, medical insurance, pricing, and community care arrangements, the stronger reliance on regulatory instruments at the national level also reflects the need to reduce interdepartmental coordination uncertainty and clarify responsibilities before broader policy expansion (22, 23). Under such conditions, the national preference for regulatory tools may be understood as a form of institutional risk control rather than a purely technical policy choice.

By contrast, local governments face more immediate pressure to respond to aging-related care needs and to translate broad policy directives into workable service arrangements. Their stronger reliance on pilot mechanisms and technology-support instruments reflects a pragmatic implementation logic under bounded authority. Compared with payment reform or cross-sector institutional redesign, digital platforms and operational support tools are often more feasible for local adaptation and service delivery. In this sense, the observed divergence in policy structure also mirrors China's broader “pilot first, then standardize” governance trajectory, in which local experimentation supports model refinement and service implementation, while national policy remains more focused on rule consolidation and regulatory stabilization (20, 22, 24).

This cross-level tension is not unique to China, but China presents a more structured institutional form of a broader digital health governance challenge. In countries such as the United Kingdom and Australia, the expansion of digitally enabled health and care services has likewise required governments to balance national standard-setting with local implementation capacity, particularly in areas such as remote monitoring, care coordination, and service integration. Recent policy developments in these settings show that digital care expansion depends not only on technological uptake, but also on the alignment of governance, pricing, and cross-sector coordination arrangements. However, compared with systems in which service integration is shaped more strongly by purchaser–provider negotiation or decentralized commissioning, the Chinese case is more explicitly organized through hierarchical governance and pilot-based scaling. As a result, a broadly shared governance challenge is expressed in China in a more distinct national–local pattern, in which central policies prioritize regulatory consolidation while local policies assume greater responsibility for implementation adaptation and service translation (25, 26).

### 4.2 Vertical disparities in policy instrument configuration and structural mismatches in internet plus nursing service

Against this institutional background, the divergence in policy instrument configuration was not accidental, but reflected different governance priorities and implementation constraints across levels (27). Although both national and local policies are predominantly characterized by environmental instruments, significant differences exist in their internal composition and evolutionary trajectories. At the national level, there has been a continuous reinforcement of standards, norms, and regulatory oversight. The declining proportion of pilot instruments over time clearly indicates that the governance focus of “Internet Plus Nursing Services” has shifted from innovation authorization toward institutional convergence. At the local level, however, a distinct supply-side orientation persists, with intelligent technology support, service capacity building, and pilot promotion mechanisms running through the entire policy cycle. This differentiated pattern is not merely a neutral division of functional labor; rather, it embeds two structural mismatches that constrain policy effectiveness.

First, a mismatch between regulatory requirements and incentive compensation. At the national level, regulatory instruments for “Internet Plus Nursing Services” have continued to intensify, while the embedding of medical insurance payments, price adjustments, and performance-based incentive instruments has remained relatively modest. Consequently, local governments lack commensurate resource compensation channels when implementing regulations concerning home-visit service standards, service quality benchmarks, and information security requirements. A dynamic balance has yet to be achieved between heightened regulatory intensity and the capacity to absorb implementation costs—a issue commonly observed in the implementation of primary care and older adult care service policies (23, 28). This structural mismatch is also prominent in international digital health governance practices. In the field of telemedicine, coordination among regulatory compliance, practice qualifications, data privacy, and payment mechanisms has become a core issue. When regulatory frameworks are continuously strengthened without the simultaneous embedding of payment systems and incentive mechanisms, primary care service providers face institutional cost pressures, thereby diminishing policy implementation enthusiasm. Similarly, in nurse-led remote service practices, the misalignment between scope-of-practice definitions and reimbursement arrangements has been identified as a significant institutional barrier to scaling digital nursing services (29, 30). These experiences are highly consistent with the regulatory-incentive structural mismatch trend revealed in this study, indicating that this issue is not unique to the Chinese context but rather constitutes a common challenge in the integration process of digital health policies under multi-level governance.

Second, a mismatch between supply capacity and demand activation. In the early stages, local policies concentrated investments on platform development, nurse training, and service system construction for “Internet Plus Nursing Services,” while demand-side instruments were only introduced during the developmental phase. This temporal lag has resulted in a periodic disconnection between service supply capacity and effective demand (31, 32). When digital infrastructure development lacks accompanying demand-side payment incentives and usage guidance mechanisms, a phenomenon of “technology presence but underutilization” often emerges. Particularly among older adults, factors such as digital accessibility, affordability, and digital literacy significantly influence the actual utilization rates of digital nursing services (33). Therefore, the synchronized embedding of supply- and demand-side instruments has become a critical condition for enhancing the sustainability of digital nursing systems. This international experience further corroborates the findings of this study: when first-mover advantages in capacity building are not coupled with demand activation mechanisms, they struggle to translate into efficiency gains in matching supply with demand. The fundamental reason why the first-mover advantage in capacity building fails to translate promptly into efficiency in matching supply and demand lies in the institutional inertia of bureaucratic organizations, which prioritizes visible capacity building over the identification of latent needs. The key to narrowing this vertical gap does not lie in simply equalizing the types of instruments used at the central and local levels. Rather, it lies in constructing a complete transmission chain—from regulatory requirements to resource allocation, and from supply expansion to demand response—tailored to the specific characteristics of “Internet Plus Nursing Services.”

### 4.3 Common shortcomings in policy design consistency and leverage points for improvement in internet plus nursing service

The variations in the topographic patterns of the PMC index serve as a visual representation of the coordination degree among the design dimensions of “Internet Plus Nursing Services” policies. High-scoring policies exhibit a balanced distribution of scores across dimensions, reflecting intrinsic coherence among policy objectives, instrument mixes, and evaluation arrangements. In contrast, low-scoring policies display pronounced depressions in specific dimensions, indicating a lack of effective functional integration among design elements. Of greater significance for policy improvement is the recurrence of commonly weak dimensions across governance levels: both policy coverage scope and policy evaluation mechanisms register low scores at the national and local levels, constituting a shared bottleneck constraining the effectiveness enhancement of “Internet Plus Nursing Services” policies.

More importantly, the PMC results suggest that the observed weaknesses should not be understood as isolated deficiencies in one or two dimensions alone. Rather, they reflect a broader pattern of structural imbalance that becomes more pronounced at the local level. While national policies generally maintain stronger consistency in framework-oriented dimensions, local policies more often display a configuration in which implementation-oriented elements are relatively well developed but dimensions related to long-term effectiveness, integrated goal formation, and evaluation feedback remain comparatively under-specified. In this sense, the key issue is not simply lower scores on indicators, but uneven development across different dimensions of policy design maturity.

The ambiguity in policy coverage scope should not be understood merely as a technical omission, but rather as a governance outcome shaped by the interaction between dual institutional logics and differentiated responsibilities across government levels. At the national level, “Internet Plus Nursing Services” are framed more strongly within the regulatory logic of the medical service system, which emphasizes standardization, patient safety, institutional qualification, and boundary control (32). By contrast, at the local level, policy design is more directly influenced by the service delivery logic of older adult care and community-based support, which prioritizes accessibility, continuity, and responsiveness to older adults with limited mobility (34). This interpretation is also consistent with the present study's text-analytic findings, which show that national policies are more strongly concentrated around governance- and regulation-related themes, whereas local policies are more closely aligned with older adult care, community-based support, and home-service implementation. In this sense, the tension between regulatory clarity and service inclusiveness does not manifest uniformly across levels, but appears at the national level mainly as a preference for institutional authorization and boundary definition, while at the local level it is expressed more as implementation-oriented flexibility and broader interpretive space around target populations and service items. At the same time, such ambiguity may not be entirely unintended, because recent research suggests that ambiguity in Chinese policy communication may function strategically by preserving local discretion and encouraging adaptive experimentation in complex governance settings (35). However, although such flexibility may be institutionally useful during the pilot and expansion stages, its continued presence in the phase of institutional consolidation may contribute to uneven local interpretation, inconsistent service eligibility, and fragmented implementation standards. The weakness of policy evaluation mechanisms indicates that dynamic feedback and performance assessment concerning service outcomes, patient experiences, and cost-effectiveness have not yet been embedded into the core design of “Internet Plus Nursing Services.” The current policy system remains oriented toward directive output rather than being driven by outcome feedback (36). The underlying cause of this evaluation deficit lies not in insufficient technical capacity, but in an institutional design that prioritizes ex-ante regulation over investment in ex-post learning mechanisms.

This weakness in evaluation and feedback mechanisms also reflects a broader challenge in digital health policy rather than a purely country-specific shortcoming. Recent international evidence shows that digital home care and virtual care programs often expand faster than the development of robust evaluation frameworks, with outcome measurement, user experience, and cost-effectiveness assessment remaining insufficiently embedded in routine policy adjustment. At the same time, the Chinese case gives this common problem a more specific institutional expression. In systems where policy expansion is driven more strongly by hierarchical mobilization and regulatory rollout, evaluation is more likely to remain subordinate to ex ante supervision. By contrast, in some other settings, evaluation gaps are more often associated with fragmented commissioning, funding complexity, or uneven digital infrastructure. Therefore, the weakness identified here should be understood as both a common structural challenge in digital care governance and a problem intensified by China's particular mode of policy expansion (6, 37).

These two common shortcomings anchor the priority directions for optimizing “Internet Plus Nursing Services” policies: boundary definition and feedback loops constitute the most leveraged improvement points. Clarifying the eligibility criteria for service recipients and the list of service items can provide stable expectations for local implementation. Embedding regularized evaluation mechanisms and establishing a mandatory link between evaluation outcomes and policy revisions can create a standardized channel for institutional iteration (38, 39).

From an operational governance perspective, strengthening the definition of policy scope and enhancing evaluation mechanisms carries significant implications for the implementation of Internet Plus Nursing Services. Clearly defined eligibility criteria and standardized service lists provide healthcare institutions and community-based nursing providers with stable and consistent operational benchmarks, thereby minimizing regional variation in policy interpretation and improving service uniformity. Concurrently, the integration of routine evaluation indicators—such as service utilization rates, patient satisfaction, and cost-effectiveness—enables local health authorities to identify operational constraints in a timely manner and make necessary adjustments to service delivery models. In practice, such feedback-oriented evaluation systems support evidence-based decision-making and foster the ongoing refinement of digital nursing services within the broader framework of long-term care systems.

From the perspective of policy implementation frameworks, this multidimensional imbalance may have several consequences. When long-term effectiveness is weakly articulated, implementation may remain oriented toward immediate deployment rather than sustained institutional consolidation. When evaluation arrangements remain underdeveloped, policy learning and adaptive adjustment become difficult to institutionalize. As a result, differences in local implementation may persist not only because of contextual diversity, but also because policy design has not yet fully translated implementation expansion into stable and internally coordinated governance arrangements.

### 4.4 Directional anchoring and pathway design for optimizing internet plus nursing service policies

Based on the structural gaps identified in this study, policy optimization should focus on a limited set of feasible institutional actions within the existing governance framework. A more practical direction is to strengthen the coupling among service boundaries, service entry, payment support, evaluation procedures, organizational capacity, and national–local coordination.

A necessary starting point is to reduce ambiguity in policy coverage by establishing a minimum national framework for service boundaries. As this study indicates, inconsistency is concentrated in dimensions related to policy coverage and design completeness, particularly at the local level. National health authorities should therefore define a baseline framework specifying eligible service populations, major home-visit nursing services, and key exclusion or risk conditions. Local governments may expand service content according to regional conditions, but should not fall below this national minimum standard.

Local implementation also requires a more stable mechanism for identifying and converting potential demand into actual service use. Although local policies place considerable emphasis on implementation and service delivery, the translation of service capacity into effective demand response remains insufficient. Primary healthcare institutions, family doctor teams, and community older adult care service stations could therefore be used to identify older adults with limited mobility, chronic disease follow-up needs, or post-discharge nursing demands, and to refer eligible cases directly to Internet Plus Nursing Services platforms. Integrating service entry into existing community-based health management workflows is likely to be more feasible than relying mainly on passive application by patients and families.

Service expansion is unlikely to be sustained without clearer payment and compensation arrangements. Since local implementation has progressed more rapidly than the institutionalization of supporting incentives, a practical step would be to develop a nationally recommended catalog of reimbursable home-visit nursing services together with reference pricing standards for major service categories. On this basis, local governments could adjust reimbursement levels and fiscal subsidies according to regional capacity while operating within a more stable payment framework.

Policy adjustment should likewise be anchored in routine evaluation rather than occasional administrative response. Given that policy evaluation emerged as one of the weakest dimensions in the PMC analysis, local governments should report a concise set of core indicators on a regular basis, including service volume, patient satisfaction, adverse events, and response timeliness. These data should then be incorporated into annual policy review procedures at the provincial or national level, so that recurrent implementation barriers can trigger timely revision of service lists, technical guidance, or reimbursement rules.

Finally, these measures should be supported by stronger organizational capacity and a more regularized national–local feedback interface. Pilot areas could designate coordinating units within medical institutions or community health centers to manage referral, scheduling, staff deployment, and follow-up documentation. At the same time, local governments should submit standardized annual reports on service uptake, implementation barriers, safety risks, and policy adjustment needs, while national authorities should use these reports to update technical guidance and regulatory rules on a periodic basis. Together, these arrangements would help shift the policy system from one-way transmission toward a more stable process of feedback, adjustment, and iterative improvement.

### 4.5 Strengths and limitations

From a methodological perspective, the three-dimensional framework developed in this study offers clear value: by integrating policy themes, policy instruments, and design consistency within a single analytical structure, it enables a more comprehensive diagnosis of policy structure than single-method approaches. Keyword analysis captures shifts in policy attention and cross-level thematic orientation; policy instrument coding reveals how governance tools are configured and distributed; and PMC evaluation assesses whether these elements remain internally coordinated and balanced at the policy design level. Together, these dimensions identify structural problems that may be difficult to detect through any one method alone—such as misalignment between policy priorities, instrument allocation, and design coherence. Thus, the framework not only strengthens the explanatory depth of the present study but also provides a transferable analytical approach for other digital health policy domains characterized by multi-level governance, including telehealth, internet hospitals, and home-based digital care.

The findings also carry broader implications for governance, public policy, and policy practice. Rather than viewing digital nursing policy as a simple extension of technological adoption, the results suggest that it should be understood as a process of institutional coordination across governance levels and policy subsystems. The divergence between national and local policies indicates that digital health governance is shaped not only by vertical divisions of responsibility, but also by the alignment of regulatory arrangements, financing mechanisms, and service delivery structures. More broadly, policy design quality in digital health governance depends on the structural coupling among thematic priorities, policy tools, and institutional feedback arrangements. These findings further suggest that the sustainable development of digital nursing services requires policy design to move beyond initial expansion toward greater internal coherence, stronger cross-sector coordination, and more explicit evaluation and feedback arrangements. For local pilot programs in particular, a shift from short-term implementation targets toward evidence-based performance assessment may support longer-term institutional learning and improve the overall coherence of digital nursing policy.

At the same time, several limitations should be acknowledged. First, because this study is based primarily on policy texts, it is more suitable for examining policy design, institutional orientation, and structural consistency than for directly assessing implementation effects, service quality, or health outcomes. Second, although the integration of three analytical dimensions improves interpretive depth, the framework remains essentially text-centered and is therefore less capable of capturing informal negotiation, interdepartmental bargaining, local adaptation, and dynamic feedback processes during implementation. Third, each component of the framework has its own analytical blind spots: keyword co-occurrence reflects textual salience rather than actual policy priority, policy instrument coding inevitably involves rule-based judgment, and PMC scoring, while useful for revealing balance across dimensions, may simplify contextual complexity by translating policy quality into standardized indicators. Finally, because the analysis is situated within China's institutional context, caution is needed when extending either the substantive findings or the framework itself directly to other health systems. Future research could combine policy text analysis with implementation data, service utilization indicators, reimbursement records, and stakeholder interviews to further test the explanatory scope and practical applicability of this framework.

## 5 Conclusion

This study showed that China's “Internet Plus Nursing Services” policies exhibit clear hierarchical differentiation across national and local levels in terms of policy themes, instrument configuration, and design consistency. National policies were more strongly oriented toward regulatory coordination, institutional standardization, and system governance, whereas local policies placed greater emphasis on implementation arrangements, service delivery, and community-based care. Although environment-oriented instruments remained dominant at both levels, important structural imbalances persisted, particularly in the alignment between regulatory requirements and incentive arrangements, the coordination among policy instruments, and the development of evaluation and feedback mechanisms. These findings suggest that, while the policy system has achieved substantial institutional expansion, its internal coordination and design balance remain insufficient, especially in local policy design.

Future policy development should therefore place greater emphasis on strengthening incentive compatibility, improving the integration of supply-, demand-, and environment-oriented instruments, and embedding more explicit evaluation, feedback, and adaptive adjustment mechanisms into the policy process. Beyond its empirical findings, this study also contributes methodologically by constructing a three-dimensional analytical framework that integrates policy themes, policy instruments, and PMC-based evaluation. By linking policy structure, implementation logic, and design consistency within a unified analytical framework, it provides a reusable analytical paradigm for the structural diagnosis of digital health policies and helps identify governance bottlenecks and design weaknesses that may be difficult to capture through single-method approaches. In this sense, the study not only deepens understanding of the policy evolution of “Internet Plus Nursing Services” in China, but also enhances the theoretical generalizability of the research and offers a transferable analytical approach for other digital health policy domains.
