# Supplementary material for: Hierarchical differentiation and design gaps in China's Internet Plus Nursing Services Policies: a PMC index analysis
Source: Front Public Health. 2026 May 8;14:1829126. doi: 10.3389/fpubh.2026.1829126 (PMC13194111; doi:10.3389/fpubh.2026.1829126)
Supplement: Supplementary file 1 [file Table_1.docx]

Supplementary Material 1

Supplementary Material 1 presents the complete list of policy documents included in this study. The identification, screening, and eligibility assessment procedures are detailed in the Methods section and illustrated in Figure 1 of the main text. A total of 123 policy documents issued between 2018 and 2025 met the predefined inclusion criteria and were retained for analysis.

**Table S1.** National-Level Internet-Enabled Nursing Service Policy Documents Included in the Study. (n = 20)

| NO. | Policy Document | Issuing Date | Issuing Department |
| --- | --- | --- | --- |
| 1 | Opinions of the General Office of the State Council on Promoting the Development of "Internet Plus Healthcare" | 2018.04 | General Office of the State Council |
| 2 | Guiding Opinions on Promoting the Reform and Development of the Nursing Service Industry | 2018.07 | National Health Commission |
| 3 | Administrative Measures for Internet-based Diagnosis and Treatment (Interim) | 2018.07 | National Health Commission |
| 4 | Notice of the General Office of the National Health Commission on Carrying Out the Pilot Work of "Internet Plus Nursing Services" | 2019.01 | National Health Commission |
| 5 | Guiding Opinions of the National Healthcare Security Administration on Improving the Pricing and Health Insurance Payment Policies for "Internet Plus" Medical Services | 2019.08 | National Healthcare Security Administration |
| 6 | Notice of the General Office of the National Health Commission on Further Promoting the Pilot Work of "Internet Plus Nursing Services" | 2020.12 | National Health Commission |
| 7 | Notice on Further Advancing the "Five Ones" Service Action for "Internet Plus Healthcare" | 2020.12 | National Health Commission |
| 8 | Notice of the National Health Commission on Issuing the National Nursing Development Plan (2021-2025) | 2021.05 | Department of Medical Administration |
| 9 | Notice of the General Office of the State Council on Issuing the 14th Five-Year Plan for Universal Healthcare Security | 2021.09 | General Office of the State Council |
| 10 | Notice on Issuing the Action Plan for the Development of the Smart Health and Elderly Care Industry (2021-2025) | 2021.10 | National Health Commission and two other departments |
| 11 | Notice of the General Office of the State Council on Issuing the 14th Five-Year Plan for National Health | 2022.04 | General Office of the State Council |
| 12 | Notice on Further Improving Direct Settlement for Off-site Medical Treatment Across Provinces Under Basic Medical Insurance | 2022.06 | National Healthcare Security Administration |
| 13 | Guiding Opinions on Further Advancing the Integrated Development of Medical and Elderly Care Services | 2022.07 | National Health Commission and 11 other relevant authorities |
| 14 | Notice on Issuing the Action Plan for Further Improving Nursing Services (2023-2025) | 2023.06 | National Health Commission and National Administration of Traditional Chinese Medicine |
| 15 | Notice on Implementing Properly the Work of Basic Public Health Service Programs for 2023 | 2023.07 | National Health Commission and 3 other relevant authorities |
| 16 | Notice of the National Healthcare Security Administration on Issuing the Guiding Principles for Project Establishment of Comprehensive Diagnosis and Examination Pricing Items for Medical Services (Interim) | 2024.11 | National Healthcare Security Administration |
| 17 | Guiding Opinions on Promoting High-Quality Development of Medical and Elderly Care Integration Services | 2024.12 | National Health Commission and 5 other relevant authorities |
| 18 | Notice on Implementing Practical Projects for Public Benefit by the Healthcare System in 2025 | 2025.02 | National Health Commission, the National Administration of Traditional Chinese Medicine and the National Disease Control and Prevention |
| 19 | Notice of the National Health Commission on Issuing the Work Plan for Demonstration Projects on the Integration of Medical and Elderly Care Services | 2025.07 | National Health Commission |
| 20 | Notice on Issuing the Action Plan for Enhancing Geriatric Nursing Service Capacity | 2025.12 | National Health Commission and 3 other relevant authorities |

**Table S2.** Beijing Municipal Internet-Enabled Nursing Service Policy Documents Included in the Study. (n = 16)

| NO. | Policy Document | Issuing Date | Issuing Department |
| --- | --- | --- | --- |
| 1 | **Notice of Beijing Municipal Health Commission, Beijing Municipal Market Supervision Administration and Beijing Municipal Healthcare Security Administration on Developing and Regulating Internet-based Home Care Services (Jing Wei Yi [2018] No. 214)** | 2018.12 | **Beijing Municipal Health Commission, Beijing Municipal Market Supervision Administration and Beijing Municipal Healthcare Security Administration** |
| 2 | **Notice on Issuing the Implementation Plan for Promoting Reform and Development of the Nursing Service Industry in Beijing** | 2019.07 | **Beijing Municipal Health Commission** |
| 3 | **Notice of Beijing Municipal Health Commission on Issuing the Key Work Points of Geriatric Health in Beijing for 2020** | 2020.03 | Beijing Municipal Health Commission |
| 4 | **Notice of Beijing Municipal Health Commission on Issuing the Key Work Points of Primary Health in Beijing for 2020** | 2020.06 | Beijing Municipal Health Commission |
| 5 | **Implementation Plan of Beijing Municipality for Establishing and Improving the Geriatric Health Service System** | 2020.12 | Beijing Municipal Health Commission |
| 6 | **Notice of Beijing Municipal Health Commission on Issuing the Key Work Points of Geriatric Health in Beijing for 2021** | 2021.03 | Beijing Municipal Health Commission |
| 7 | **Notice of Beijing Municipal Health Commission and Beijing Municipal Civil Affairs Bureau on Carrying Out the Action for Quality Improvement of Medical and Health Care Services in Medical and Elderly Care Integration Institutions in 2021** | 2021.06 | Beijing Municipal Health Commission and **Beijing Municipal Civil Affairs Bureau** |
| 8 | **Notice of Beijing Municipal Health Commission on Issuing the Action Plan for Improving Medical Services in Beijing for 2022** | 2022.06 | Beijing Municipal Health Commission |
| 9 | **Work Plan of Beijing Municipality for the Pilot Program of Geriatric Medical and Nursing Services** | 2022.09 | Beijing Municipal Health Commission |
| 10 | **Notice of Beijing Municipal Health Commission on Properly Carrying Out the Transformation and Construction of Hospice Care Centers and Geriatric Nursing Centers** | 2023.05 | Beijing Municipal Health Commission |
| 11 | **Notice of Beijing Municipal Health Commission on Issuing the Evaluation Criteria for Age-friendly Medical Institutions in Beijing (2023 Edition)** | 2023.07 | Beijing Municipal Health Commission |
| 12 | **Notice of Beijing Municipal Health Commission on Issuing the Community Geriatric Health Service Specifications in Beijing (2023 Edition)** | 2023.11 | Beijing Municipal Health Commission |
| 13 | **Notice of Beijing Municipal Health Commission on Issuing the Work Plan for Improving Medical Services in Beijing for 2024** | 2024.03 | Beijing Municipal Health Commission |
| 14 | **Notice of the General Office of Beijing Municipal People's Government on Issuing the Beijing Action Plan for Accelerating Collaborative Innovation in Medicine and Health (2024-2026)** | 2024.05 | **General Office of Beijing Municipal People's Government** |
| 15 | **Notice of Beijing Municipal Health Commission on Issuing the Work Plan for Formulating the Healthy Beijing Initiative During the 15th Five-Year Plan Period** | 2025.01 | Beijing Municipal Health Commission |
| 16 | **Notice of Beijing Municipal Health Commission and Four Other Departments on Carrying Out Action to Promote Medical and Elderly Care Integration and Facilitating High-Quality Development of Integrated Medical and Elderly Care Services** | 2025.12 | Beijing Municipal Health Commissionand 4 other relevant authorities |

**Table S3.** Tianjin Municipal Internet-Enabled Nursing Service Policy Documents Included in the Study. (n = 19)

| NO. | Policy Document | Issuing Date | Issuing Department |
| --- | --- | --- | --- |
| 1 | **Tianjin Smart Healthcare and Health Action Plan Implementation Opinions on Strengthening the Work of Home-Based Elderly Care Services** | 2018.08 | **Tianjin Municipal Bureau of Industry and Information Technology** |
| 2 | **Implementation Opinions of the General Office of Tianjin Municipal People's Government on Promoting the Development of "Internet Plus Healthcare"** | 2018.11 | **General Office of Tianjin Municipal People's Government** |
| 3 | **Implementation Plan for the Pilot Work of "Internet Plus Nursing Services" in Tianjin** | 2019.04 | **Tianjin Municipal Health Commission** |
| 4 | **Core Policies of Tianjin Municipality for Convenience and Benefit of the People via "Internet Plus Healthcare" (2019)** | 2019.05 | **Tianjin Municipal Health Commission** |
| 5 | **Notice of the Municipal Healthcare Security Administration, Municipal Health Commission and Municipal Human Resources and Social Security Bureau on Regulating the Pricing and Health Insurance Payment Policies for "Internet Plus" Medical Services** | 2020.02 | **Tianjin Municipal Healthcare Security Administration, Tianjin Municipal Health Commission and Tianjin Municipal Human Resources and Social Security Bureau** |
| 6 | **Implementation Guidelines for the Construction of Digital Health Consortia in Primary Medical and Health Institutions in Tianjin** | 2020.04 | **Tianjin Municipal Health Commission** |
| 7 | **Notice on Issuing the Administrative Measures for Health Insurance Payment of "Internet Plus" Medical Services in Tianjin (Interim)** | 2020.10 | **Tianjin Municipal Healthcare Security Administration** |
| 8 | **Pilot Implementation Plan for the Long-Term Care Insurance System in Tianjin (2020)** | 2020.12 | **General Office of Tianjin Municipal People's Government** |
| 9 | **Notice of Tianjin Municipal Health Commission on Issuing the 14th Five-Year Plan for the Development of Primary Health in Tianjin** | 2021.06 | **Tianjin Municipal Health Commission** |
| 10 | **14th Five-Year Plan for the Development of Primary Health in Tianjin (2021-2025)** | 2021.07 | **Tianjin Municipal Health Commission** |
| 11 | **Action Plan for the Development of Home-Based Elderly Care Services in Tianjin (2021-2025)** | 2021.10 | **Tianjin Municipal Civil Affairs Bureau** |
| 12 | **Specific Measures of the General Office of Tianjin Municipal People's Government on Further Advancing the Development of Elderly Care Programs and the Construction of the Elderly Care Service System** | 2022.07 | **General Office of Tianjin Municipal People's Government** |
| 13 | **The 14th Five-Year Plan for the Development of the Elderly Care Service System in Tianjin and the Long-Range Objectives Through the Year 2035** | 2022.10 | **Tianjin Municipal Civil Affairs Bureau** |
| 14 | **Notice of the General Office of Tianjin Municipal People's Government on Issuing the Implementation Plan for Further Developing the Long-Term Care Insurance System Pilot in Tianjin** | 2022.12 | **General Office of Tianjin Municipal People's Government** |
| 15 | **Action Plan for Further Improving Medical and Nursing Services in Tianjin (2023-2025)** | 2023.07 | **Tianjin Municipal Health Commission** |
| 16 | **Implementation Plan of Tianjin Municipality for Promoting the Construction of a Basic Elderly Care Service System (2023 Edition)** | 2023.08 | **General Office of Tianjin Municipal People's Government** |
| 17 | **Notice of the General Office of Tianjin Municipal People's Government on Issuing the Implementation Plan for Developing the Silver Economy and Enhancing the Welfare of the Elderly in Tianjin** | 2024.10 | **General Office of Tianjin Municipal People's Government** |
| 18 | **Notice of Tianjin Municipal Health Commission and Tianjin Municipal Disease Control and Prevention Bureau on Issuing 16 Measures for Health Services for Public Benefit in Tianjin in 2025** | 2025.01 | **Tianjin Municipal Health Commission and Tianjin Municipal Disease Control and Prevention Bureau** |
| 19 | **Implementation Opinions of Tianjin Municipal People's Government on Deepening the Reform and Development of Elderly Care Services** | 2025.10 | **General Office of Tianjin Municipal People's Government** |

**Table S4.** Shanghai Municipal Internet-Enabled Nursing Service Policy Documents Included in the Study. (n = 18)

| NO. | Policy Document | Issuing Date | Issuing Department |
| --- | --- | --- | --- |
| 1 | **Notice of the General Office of Shanghai Municipal People's Government on Issuing the Administrative Measures for Unified Needs Assessment and Services for Senior Care in Shanghai** | 2018.01 | **Shanghai Municipal People's Government** |
| 2 | **Notice on Issuing the Implementation Plan for Nursing Development in Shanghai (2018-2020)** | 2018.04 | **Shanghai Municipal Health Commission** |
| 3 | **Notice on Issuing the Implementation Plan for the Pilot Work of "Internet Plus Nursing Services" in Shanghai** | 2019.07 | **Shanghai Municipal Health Commission** |
| 4 | **Notice of Shanghai Municipal People's Government on Issuing the Implementation Opinions on Promoting the Healthy Shanghai Initiative** | 2019.08 | **Shanghai Municipal People's Government** |
| 5 | **Notice on Issuing the Key Work Points of Shanghai Senior Care Services for 2020** | 2020.03 | **Shanghai Municipal Civil Affairs Bureau** |
| 6 | **Notice of Shanghai Municipal Human Resources and Social Security Bureau and Eight Other Departments on Issuing the Implementation Opinions on Strengthening the Development of the Elderly Care Worker Workforce and Improving the Quality of Elderly Care Services** | 2020.05 | **Shanghai Municipal Human Resources and Social Security Bureau and eight other departments** |
| 7 | **Notice on Issuing the Implementation Plan for the 2020 Special Action on Improving Service Quality in Elderly Care Institutions** | 2020.06 | **Shanghai Municipal Civil Affairs Bureau** |
| 8 | **Notice of Shanghai Municipal Civil Affairs Bureau on Comprehensively Promoting Aging-Friendly Home Modification in Shanghai** | 2021.08 | **Shanghai Municipal Civil Affairs Bureau** |
| 9 | **Notice of Shanghai Municipal Civil Affairs Bureau on Issuing the Administrative Measures for Community Senior Day Care Institutions in Shanghai** | 2021.12 | **Shanghai Municipal Civil Affairs Bureau** |
| 10 | **Implementation Opinions of the General Office of Shanghai Municipal People's Government on Promoting the Construction of Elderly Care Facilities in Shanghai During the 14th Five-Year Plan Period** | 2021.12 | **Shanghai Municipal People's Government** |
| 11 | **Notice of Shanghai Municipal Civil Affairs Bureau on Issuing the Shanghai Three-Year Action Plan for Promoting the Construction of Smart Senior Care Homes (2023-2025)** | 2022.12 | **Shanghai Municipal Civil Affairs Bureau** |
| 12 | **Notice of the General Office of Shanghai Municipal People's Government on Issuing the Amended Administrative Measures for Unified Needs Assessment and Services for Senior Care in Shanghai** | 2022.12 | **Shanghai Municipal Development and Reform Commission** |
| 13 | **Administrative Measures for Designated Nursing Service Institutions Under Long-Term Care Insurance in Shanghai (Interim)** | 2023.02 | **Shanghai Municipal People's Government** |
| 14 | **Notice on Issuing the List of Basic Elderly Care Services in Shanghai (2023 Edition)** | 2023.04 | **Shanghai Municipal Civil Affairs Bureau** |
| 15 | **Notice on Issuing the List of Basic Elderly Care Services in Shanghai (2024 Edition)** | 2024.04 | **Shanghai Municipal Civil Affairs Bureau** |
| 16 | **Notice of the General Office of Shanghai Municipal People's Government on Issuing the Shanghai Action Plan for Advancing Aging Technology Innovation Development (2024-2027)** | 2024.07 | **Shanghai Municipal People's Government** |
| 17 | **Notice of Shanghai Municipal Civil Affairs Bureau on Issuing the Three-Year Action Plan for Quality Improvement of Elderly Care Institutions in Shanghai (2025-2027)** | 2025.01 | **Shanghai Municipal People's Government** |
| 18 | **Shanghai List of Basic Elderly Care Services (2025 Edition)** | 2025.04 | **Shanghai Municipal Civil Affairs Bureau** |

**Table S5.** Jiangsu Provincial Internet-Enabled Nursing Service Policy Documents Included in the Study. (n = 18)

| NO. | Policy Document | Issuing Date | Issuing Department |
| --- | --- | --- | --- |
| 1 | **Notice of the General Office of Jiangsu Provincial People's Government on Issuing the 13th Five-Year Plan for Health and the Construction of a Modern Healthcare System in Jiangsu Province** | 2018.01 | **General Office of Jiangsu Provincial People's Government** |
| 2 | **Notice of the General Office of Jiangsu Provincial People's Government on Forwarding the Implementation Opinions of the Provincial Health and Family Planning Commission and Other Departments on Deepening the Integration of Medical and Health Services with Elderly Care Services** | 2018.03 | **General Office of Jiangsu Provincial People's Government** |
| 3 | **Notice on Issuing the Implementation Plan for the Pilot Work of "Internet Plus Nursing Services" in Jiangsu Province** | 2019.04 | **Jiangsu Provincial Health Commission** |
| 4 | **Notice of Jiangsu Provincial Health Commission on Comprehensively Launching Activities for the Convenience and Benefit of the People via "Internet Plus Healthcare" (Su Wei Gui Hua [2018] No. 39)** | 2019.07 | **Jiangsu Provincial Health Commission** |
| 5 | **Notice of the General Office of Jiangsu Provincial People's Government on Issuing the Implementation Plan for Advancing the Healthy China Initiative and Building a Healthy Jiangsu** | 2020.02 | **General Office of Jiangsu Provincial People's Government** |
| 6 | **Several Measures on Deepening the Integration of Medical and Elderly Care Services** | 2020.11 | **Jiangsu Provincial Health Commission** |
| 7 | **Notice of the General Office of Jiangsu Provincial People's Government on Issuing the 14th Five-Year Plan for Medical Security Development in Jiangsu Province** | 2021.09 | **General Office of Jiangsu Provincial People's Government** |
| 8 | **Implementation Opinions on Establishing and Improving a Comprehensive Supervision System for Elderly Care Services and Promoting High-Quality Development of Elderly Care Services** | 2021.10 | **General Office of Jiangsu Provincial People's Government** |
| 9 | **Notice of the General Office of Jiangsu Provincial People's Government on Issuing the 14th Five-Year Plan for the Development of Elderly Care Services in Jiangsu Province** | 2021.12 | **General Office of Jiangsu Provincial People's Government** |
| 10 | **Notice on Issuing the Jiangsu Provincial Guidance List of Basic Elderly Care Services (2022 Edition)** | 2022.06 | **Jiangsu Provincial Department of Civil Affairs** |
| 11 | **Reply to Proposal No. 0091 of the Fourth Session of the 12th Jiangsu Provincial Committee of the Chinese People's Political Consultative Conference (CPPCC)** | 2022.07 | **Jiangsu Provincial Department of Civil Affairs** |
| 12 | **Regulations of Jiangsu Province on Elderly Care Services (2022 Revision)** | 2022.09 | **The 13th Standing Committee of the Jiangsu Provincial People's Congress** |
| 13 | **Implementation Opinions of the Jiangsu Provincial People's Government on Promoting the Development of Elderly Care Initiatives and Industry and Improving the Quality of Elderly Care Services** | 2023.05 | **General Office of Jiangsu Provincial People's Government** |
| 14 | **Implementation Plan for Further Improving the Healthcare Service System** | 2024.01 | **General Office of Jiangsu Provincial People's Government** |
| 15 | **Implementation Plan for the "Caring Meal Assistance" Special Action in Jiangsu Province** | 2024.03 | **Jiangsu Provincial Department of Civil Affairs** |
| 16 | **Notice on Issuing the Implementation Plan for Promoting High-Quality Development of the Silver Economy** | 2024.09 | **General Office of Jiangsu Provincial People's Government** |
| 17 | **Notice of Jiangsu Provincial Department of Civil Affairs on Issuing the Provincial Key Work Points of Civil Affairs for 2025** | 2025.03 | **Jiangsu Provincial Department of Civil Affairs** |
| 18 | **Notice of the General Office of Jiangsu Provincial People's Government on Issuing Several Measures for Implementing Special Actions to Boost Consumption in Jiangsu Province** | 2025.05 | **General Office of Jiangsu Provincial People's Government** |

**Table S6.** Zhejiang Provincial Internet-Enabled Nursing Service Policy Documents Included in the Study. (n = 16)

| NO. | Policy Document | Issuing Date | Issuing Department |
| --- | --- | --- | --- |
| 1 | **Implementation Opinions of the General Office of Zhejiang Provincial People's Government on Deepening Comprehensive Reform of Elderly Care Services and Improving Service Quality** | 2018.08 | **Zhejiang Provincial Department of Civil Affairs** |
| 2 | **Notice of Zhejiang Provincial Health Commission on Issuing the Implementation Plan for the Pilot Work of "Internet Plus Nursing Services" in Zhejiang Province (Interim)** | 2019.05 | **Zhejiang Provincial Health Commission** |
| 3 | **Implementation Opinions on Establishing the Benefit Determination and Normal Adjustment Mechanism for Basic Pensions for Urban and Rural Residents** | 2019.10 | **Zhejiang Provincial Department of Human Resources and Social Security** |
| 4 | **Implementation Opinions on Promoting the Improvement of Quality and Expansion of Capacity in the Domestic Service Industry** | 2020.02 | **General Office of Zhejiang Provincial People's Government** |
| 5 | **Notice of Zhejiang Provincial Health Commission on Issuing the Provincial Key Work Points of Health for 2020** | 2020.03 | **Zhejiang Provincial Health Commission** |
| 6 | **Notice of the General Office of Zhejiang Provincial People's Government on the Action Plan for Promoting the "New Digital Life Services" Initiative** | 2020.09 | **General Office of Zhejiang Provincial People's Government** |
| 7 | **Notice of Zhejiang Provincial Health Commission on Issuing the Implementation Plan for the Quality Improvement Action of Medical and Elderly Care Integration Institutions in Zhejiang Province (2021-2023)** | 2021.04 | **Zhejiang Provincial Health Commission** |
| 8 | **Notice of Zhejiang Provincial Health Commission on Issuing the Work Plan for Advancing Digital Reform in Medical and Health Services to Enhance Patient Experience and Facilitate Medical Access for the Elderly in Zhejiang Province (2021-2023)** | 2021.08 | **Zhejiang Provincial Health Commission** |
| 9 | **Notice of the General Office of Zhejiang Provincial Health Commission on Issuing the Implementation Plan for the Health Service Action for Disabled Elderly in Zhejiang Province** | 2021.10 | **Zhejiang Provincial Health Commission** |
| 10 | **Notice of Zhejiang Provincial Health Commission on Issuing the Implementation Plan for Further Promoting the Application of "Zheli Nursing" in Zhejiang Province** | 2022.12 | **Zhejiang Provincial Health Commission** |
| 11 | **Implementation Opinions of the General Office of Zhejiang Provincial People's Government on Accelerating the Establishment of a Basic Elderly Care Service System** | 2022.12 | **General Office of Zhejiang Provincial People's Government** |
| 12 | **Notice of the General Office of Zhejiang Provincial People's Government on Issuing the Implementation Plan for Establishing a Multi-Tiered Long-Term Care Protection System in Zhejiang Province** | 2023.12 | **General Office of Zhejiang Provincial People's Government** |
| 13 | **Notice of the General Office of Zhejiang Provincial Health Commission on Issuing the Fourth Batch of "Internet Plus Nursing Services" Programs in Zhejiang Province (Zhe Wei Ban [2024] No. 7)** | 2024.07 | **Zhejiang Provincial Health Commission** |
| 14 | **Notice of the General Office of Zhejiang Provincial Health Commission on Issuing the Implementation Plan for the Health Service Action for Disabled Elderly in Zhejiang Province** | 2024.10 | **Zhejiang Provincial Health Commission** |
| 15 | **Implementation Opinions of the General Office of Zhejiang Provincial People's Government on Developing the Silver Economy to Enhance the Welfare of the Elderly** | 2024.12 | **General Office of Zhejiang Provincial People's Government** |
| 16 | **Implementation Opinions on Strengthening the Development of the Talent Workforce in the Elderly Care Sector** | 2025.02 | **Zhejiang Provincial Department of Civil Affairs** |

**Table S7.** Guangdong Provincial Internet-Enabled Nursing Service Policy Documents Included in the Study. (n = 16)

| NO. | Policy Document | Issuing Date | Issuing Department |
| --- | --- | --- | --- |
| 1 | **Implementation Opinions of the General Office of Guangdong Provincial People's Government on Comprehensively Opening Up the Elderly Care Service Market and Improving Service Quality** | 2018.01 | **General Office of Guangdong Provincial People's Government** |
| 2 | **Notice of the General Office of Guangdong Provincial People's Government on Issuing the Action Plan for Promoting the Development of "Internet Plus Healthcare" in Guangdong Province (2018-2020)** | 2018.06 | **General Office of Guangdong Provincial People's Government** |
| 3 | **Notice on Issuing the Implementation Plan for the Pilot Work of "Internet Plus Nursing Services" in Guangdong Province** | 2019.04 | **Guangdong Provincial Health Commission** |
| 4 | **Notice of the General Office of Guangdong Provincial People's Government on Issuing Several Measures for Accelerating the Development of Elderly Care Services in Guangdong Province** | 2019.11 | **General Office of Guangdong Provincial People's Government** |
| 5 | **Notice on Issuing the Detailed Rules for the Administration of Subsidies for Basic Public Health Services in Guangdong Province** | 2020.09 | **Guangdong Provincial Department of Finance** |
| 6 | **Notice on Further Strengthening the Pilot Work of "Internet Plus Nursing Services"** | 2021.06 | **Guangdong Provincial Health Commission** |
| 7 | **The 14th Five-Year Plan for the Development of Human Resources and Social Security in Guangdong Province** | 2021.11 | **General Office of Guangdong Provincial People's Government** |
| 8 | **The 14th Five-Year Plan for the Construction of the Elderly Care Service System in Guangdong Province** | 2022.01 | **Guangdong Provincial Department of Civil Affairs** |
| 9 | **Notice on Issuing the Pilot Work Plan for Geriatric Medical and Nursing Services in Guangdong Province** | 2022.04 | **Guangdong Provincial Health Commission** |
| 10 | **Notice of Guangdong Provincial Department of Civil Affairs and Five Other Departments on Issuing the Plan and Roadmap for the Elderly Care Service Standards System in Guangdong Province (2022-2026)** | 2022.09 | **Guangdong Provincial Department of Civil Affairs** |
| 11 | **Pilot Work Plan for Geriatric Medical and Nursing Services in Guangdong Province** | 2023.04 | **Guangdong Provincial Health Commission** |
| 12 | **Notice on Issuing the Implementation Plan for Promoting Health Management for the Elderly in Urban and Rural Communities through Family Doctor Contract Services to Implement the Provincial Government's Practical Projects for People's Livelihood** | 2023.04 | **Guangdong Provincial Health Commission** |
| 13 | **Notice of the General Office of Guangdong Provincial People's Government on Issuing the Implementation Plan for Further Improving the Healthcare Service System in Guangdong Province** | 2023.10 | **General Office of Guangdong Provincial People's Government** |
| 14 | **Notice on Further Strengthening Vocational Skills Training for Elderly Care Workers in the Province** | 2024.01 | **Guangdong Provincial Department of Civil Affairs** |
| 15 | **Notice on Issuing the Implementation Plan for the Health Service Action for Disabled Elderly in Guangdong Province (2024-2027)** | 2024.11 | **Guangdong Provincial Health Commission** |
| 16 | **Notice on Issuing the Implementation Plan for Promoting High-Quality Development of the Silver Economy and Enhancing the Welfare of the Elderly in Guangdong Province** | 2025.02 | **General Office of Guangdong Provincial People's Government** |
