# Supplementary material for: Hierarchical differentiation and design gaps in China's Internet Plus Nursing Services Policies: a PMC index analysis
Source: Front Public Health. 2026 May 8;14:1829126. doi: 10.3389/fpubh.2026.1829126 (PMC13194111; doi:10.3389/fpubh.2026.1829126)
Supplement: Supplementary file 2 [file Table_2.docx]

Supplementary Material 2

This Supplementary material 2 presents the coding framework and operational procedure used for the policy instrument analysis of Internet Plus Nursing Services policies. The identification and quantification of policy instruments provided the empirical basis for comparing governance arrangements across administrative levels and examining stage specific changes in policy design. In addition to describing the classification system, this appendix also clarifies the coding unit, the treatment of multi code assignment, and the inter coder reliability procedure used to improve methodological transparency.

# Development of the Coding Framework

Based on the classical policy instrument typology proposed by Rothwell and Zegveld, and adapted to the institutional characteristics of Internet-enabled nursing services, this study constructed a three-dimensional classification system consisting of supply-based, demand-based, and environment-based instruments.

Considering that Internet-enabled nursing services simultaneously involve healthcare regulation and long-term care governance, the three main categories were further refined into several sub-categories to enhance analytical precision and contextual relevance. This structure allows the framework to capture resource allocation arrangements, demand activation mechanisms, regulatory provisions, and intergovernmental coordination embedded in policy texts.

The detailed classification scheme and operational definitions of each sub-category are presented in Table S8.

**Table S8.** Classification and meanings of Internet-based nursing service policy instruments.

| Policy instrument category | Sub-category | Refined meaning and analytical interpretation |
| --- | --- | --- |
| Supply-based instruments | Financial support and funding | Direct fiscal inputs that strengthen the resource base of Internet healthcare services and enhance service provision capacity. |
|  | Human resources and capacity building | Institutional arrangements aimed at improving the availability, competency, and sustainability of healthcare and nursing personnel involved in Internet-based services. |
|  | Technological support | Policy measures that promote the development and application of information technologies to support service innovation and operational efficiency. |
|  | Institutional support for service provision | Regulatory and organizational mechanisms that enable healthcare institutions to expand and innovate Internet-based service delivery. |
| Demand-based instruments | Service utilization incentives | Policy measures designed to stimulate service uptake by increasing the attractiveness and accessibility of Internet healthcare services to users. |
|  | Insurance coverage and payment mechanisms | Financial and reimbursement arrangements that lower user costs and facilitate demand formation for Internet healthcare services. |
|  | User-oriented guidance | Informational and guidance-oriented measures that shape user awareness and acceptance of Internet healthcare services. |
| Environment-based instruments | Regulatory framework | Formal rules and institutional arrangements that define the legal boundaries and governance structure of Internet healthcare services. |
|  | Quality and safety supervision | Oversight mechanisms aimed at ensuring service quality, patient safety, and risk control in Internet healthcare delivery. |
|  | Inter-agency coordination and governance | Governance arrangements that promote coordination and coherence across administrative actors involved in Internet healthcare regulation. |
|  | Standard setting and normative guidance | Standardization measures that enhance policy coherence, implementation consistency, and operational clarity. |

# Unit of Analysis and Coding Procedure

To improve comparability across policy levels and regions, individual policy clauses rather than entire policy documents were used as the basic unit of analysis. Each clause containing explicit institutional arrangements, implementation measures, or governance mechanisms was treated as one clause level unit. Based on this rule, a total of 4,124 clause level units were identified from the full policy corpus.

Coding followed three core principles. First, a substantive principle was adopted. Clauses were coded only when they contained concrete and operational provisions rather than general advocacy or broad policy statements. Second, a functional interpretation principle was applied. When a clause involved more than one policy instrument, the dominant institutional function was used to guide interpretation; however, multi code assignment was permitted when the clause contained substantively distinct provisions corresponding to different instrument categories. Third, contextual interpretation was used to ensure that coding reflected the policy intent embedded in the broader textual context.

Accordingly, the clause level unit served as the basic analytical unit, whereas coded references were used for frequency based statistical analysis. Because one clause could generate more than one valid coding assignment, the full coding process yielded 9,336 coded references, which formed the basis of the distributional analysis reported in the main text.

All policy documents were imported into MAXQDA software for systematic coding. During formal coding, clauses were coded one by one according to semantic meaning, and the corresponding code, location, and frequency were recorded. Ambiguous cases were reviewed iteratively and resolved through discussion and consensus in order to maintain coding consistency.

# Inter coder Reliability and Rule Calibration

To enhance methodological transparency and coding reliability, an independent pilot coding procedure was conducted before full text coding. From the 4,124 clause level units identified in the policy corpus, 412 units, representing approximately 10.0%, were randomly selected for double coding by two trained researchers using the same coding manual. Because multi code assignment was permitted for clauses containing more than one policy instrument, the pilot sample generated 934 coding decisions.

Inter coder reliability was assessed using Cohen’s kappa coefficient. The kappa value was 0.842 for the first level categories and 0.791 for the subcategory level coding, indicating substantial agreement. Disagreements were concentrated mainly in clauses that simultaneously involved implementation support, regulatory requirements, and coordination arrangements. These cases were jointly reviewed, and the coding rules were further refined before formal coding of the full dataset.

In the formal coding stage, when disagreement could not be resolved directly from the wording of an individual clause, the broader policy context and dominant institutional function of the provision were used to adjudicate the final code. This calibration process helped improve conceptual consistency across governance levels and developmental stages.

# **Structure of Policy Instrument Categories**

The coding framework distinguishes three main categories of policy instruments: supply based instruments, demand based instruments, and environment based instruments. Together, these categories reflect different modes of governmental intervention in the governance of Internet Plus Nursing Services.

Supply based instruments focus on strengthening service provision capacity through resource inputs, workforce development, technological support, and service delivery conditions. Demand based instruments focus on stimulating service uptake by reducing access barriers, improving affordability, and enhancing user acceptance. Environment based instruments establish the regulatory and institutional conditions necessary for standardized, coordinated, and safe service delivery.

The detailed subcategories and their operational meanings are presented in **Table S8**. The coded references derived from this framework form the basis of the frequency based distributional analysis reported in **Tables 4** and **5** of the main text.

# Linkage to Structural Evaluation

The policy instrument coding results also informed the construction of the PMC variable system, as described in Supplementary material 3. Specifically, the identification of policy tool categories contributed to the operationalization of variables related to policy instruments, policy functions, incentive arrangements, and governance mechanisms. In this way, the coding framework not only supported the descriptive analysis of policy instrument distribution, but also provided an analytical bridge between policy instrument configuration and the multidimensional consistency evaluation conducted through the PMC index model.
