# Supplementary material for: Hierarchical differentiation and design gaps in China's Internet Plus Nursing Services Policies: a PMC index analysis
Source: Front Public Health. 2026 May 8;14:1829126. doi: 10.3389/fpubh.2026.1829126 (PMC13194111; doi:10.3389/fpubh.2026.1829126)
Supplement: Supplementary file 3 [file Table_3.docx]

Supplementary Material 3

# Methodological Overview of the PMC Index Model

The Policy Modeling Consistency index model, originally proposed by Ruiz Estrada, is a multidimensional policy evaluation framework designed to assess the internal structural consistency of policy documents. The model enables systematic extraction of policy information from original texts and supports horizontal comparison across policy samples through a unified indicator system. In this study, the PMC index was used to evaluate the structural completeness, internal coherence, and design balance of Internet Plus Nursing Services policies at the national and local levels. The application of the model involved four sequential steps: construction of the variable system, indicator assignment and score calculation, evaluation grade classification, and three dimensional surface visualization.

# **Variable System and Indicator Assignment**

Building upon Ruiz Estrada’s framework and drawing on prior empirical applications of the PMC model, this study constructed a variable system tailored to the policy context of Internet Plus Nursing Services. A total of nine first level variables, X1 to X9, were defined, covering policy nature, policy instruments, policy effectiveness, policy scope, policy objectives, policy goals, policy functions, incentives and constraints, and policy evaluation. To improve analytical specificity, 35 second level variables were operationalized on the basis of policy text characteristics, keyword analysis, and the policy instrument coding results reported in **Supplementary material 2**.

All second level variables were assigned equal weights to maintain transparency and comparability. A binary scoring approach was adopted, whereby a value of 1 was assigned when the policy text explicitly and substantively addressed a given indicator, and 0 otherwise. The full structure of the PMC variable system, together with the first level and second level indicators and their corresponding evaluation criteria, is presented in Table 1 of the main text.

# **PMC Index Calculation and Evaluation Ctriteria**

The second step involved calculation of the PMC index. The computation of the PMC index for each policy text was conducted in three stages: assignment of second level indicators, aggregation of first level variables, and calculation of the overall PMC index. Formulae 1 and 2 were used to derive the values of second level variables, Formula 3 was used to obtain the values of first level variables, and Formula 4 was used to calculate the PMC index for each policy.For interpretive purposes, PMC scores were classified into four categories: perfect, excellent, qualified, and low. The corresponding evaluation criteria are presented in **Table S9**. In the formulae, t denotes the first level variable and j denotes the second level variable.

$$\begin{aligned} \mathbf{X}\mathbf{-N}\left[ \mathbf{0,1} \right]\#(1) \end{aligned}$$

$$\begin{aligned} \mathbf{X}\mathbf{=}\left\{ \mathbf{X}\mathbf{R}\left[ \mathbf{0,1} \right] \right\}\#(2) \end{aligned}$$

$$\begin{aligned} \boldsymbol{X}_{\boldsymbol{t}}\boldsymbol{=}\left( \sum_{\boldsymbol{j-1}}^{\boldsymbol{n}} \frac{\boldsymbol{X}_{\boldsymbol{tj}}}{\boldsymbol{T}\left( \boldsymbol{X}_{\boldsymbol{tj}} \right)} \right)\boldsymbol{t=1,2,3,...,\infty}\boldsymbol{\#(3)} \end{aligned}$$

**Table S9.** Policy PMC index evaluation level.

| PMC Score | 9 | 7.00-8.99 | 5.00-6.99 | 0-4.99 |
| --- | --- | --- | --- | --- |
| Evaluation | Perfect | Excellent | Qualified | Low |

Wherea:

t = primary variable,

j = secondary variable.

$$\begin{aligned} \boldsymbol{PMC=}\left[ \begin{aligned} \boldsymbol{X}_{\boldsymbol{1}}\left( \sum_{\boldsymbol{j=1}}^{\boldsymbol{5}} \frac{\boldsymbol{X}_{\boldsymbol{1j}}}{\boldsymbol{5}} \right)\boldsymbol{+}\boldsymbol{X}_{\boldsymbol{2}}\left( \sum_{\boldsymbol{j=1}}^{\boldsymbol{3}} \frac{\boldsymbol{X}_{\boldsymbol{2j}}}{\boldsymbol{3}} \right)\boldsymbol{+}\boldsymbol{X}_{\boldsymbol{3}}\left( \sum_{\boldsymbol{j=1}}^{\boldsymbol{4}} \frac{\boldsymbol{X}_{\boldsymbol{3j}}}{\boldsymbol{4}} \right)\boldsymbol{+} \\ \boldsymbol{X}_{\boldsymbol{4}}\left( \sum_{\boldsymbol{j=1}}^{\boldsymbol{4}} \frac{\boldsymbol{X}_{\boldsymbol{4j}}}{\boldsymbol{4}} \right)\boldsymbol{+}\boldsymbol{X}_{\boldsymbol{5}}\left( \sum_{\boldsymbol{j=1}}^{\boldsymbol{4}} \frac{\boldsymbol{X}_{\boldsymbol{5j}}}{\boldsymbol{5}} \right)\boldsymbol{+}\boldsymbol{X}_{\boldsymbol{6}}\left( \sum_{\boldsymbol{j=1}}^{\boldsymbol{5}} \frac{\boldsymbol{X}_{\boldsymbol{6j}}}{\boldsymbol{5}} \right)\boldsymbol{+} \\ \boldsymbol{X}_{\boldsymbol{7}}\left( \sum_{\boldsymbol{j=1}}^{\boldsymbol{5}} \frac{\boldsymbol{X}_{\boldsymbol{7j}}}{\boldsymbol{5}} \right)\boldsymbol{+}\boldsymbol{X}_{\boldsymbol{8}}\left( \sum_{\boldsymbol{j=1}}^{\boldsymbol{6}} \frac{\boldsymbol{X}_{\boldsymbol{8j}}}{\boldsymbol{6}} \right)\boldsymbol{+}\boldsymbol{X}_{\boldsymbol{9}}\left( \sum_{\boldsymbol{j=1}}^{\boldsymbol{2}} \frac{\boldsymbol{X}_{\boldsymbol{9j}}}{\boldsymbol{2}} \right) \end{aligned} \right]\boldsymbol{\#(4)} \end{aligned}$$

The third step involves constructing the PMC surface. The PMC surface presents the evaluation results and the relative merits of policies in the form of three-dimensional images, thereby enabling an intuitive visualization of the assessment outcomes. This study constructs a three-level matrix for policy evaluation using indicators X1 to X9, and employs the calculation method specified in Formula (5) to generate the surface diagram for each policy.

$$\begin{aligned} \mathbf{PMCsurface=}\left[ \begin{matrix} \mathbf{X}_{\mathbf{1}} & \mathbf{X}_{\mathbf{2}} & \mathbf{X}_{\mathbf{3}} \\ \mathbf{X}_{\mathbf{4}} & \mathbf{X}_{\mathbf{5}} & \mathbf{X}_{\mathbf{6}} \\ \mathbf{X}_{\mathbf{7}} & \mathbf{X}_{\mathbf{8}} & \mathbf{X}_{\mathbf{9}} \end{matrix} \right]\boldsymbol{\#(5)} \end{aligned}$$

# Selection of Policies for PMC Evaluation

To ensure the validity, comparability, and interpretability of the PMC index evaluation, a subset of representative policy documents was selected from the full policy corpus using a purposive sampling strategy. This approach is consistent with prior applications of the PMC index model, which typically focus on information-rich and structurally representative policy texts rather than the full sample.

The selection process was guided by the principle of capturing both structural diversity and institutional representativeness of “Internet Plus Nursing Services” policies. Specifically, the following criteria were applied.

First, policies were required to be highly relevant to “Internet Plus Nursing Services” or explicitly address internet-enabled, home-based, or integrated nursing care services, ensuring substantive alignment with the research focus.

Second, policies were selected across different governance levels, including national and local levels, to reflect the hierarchical structure of policy formulation and implementation. National-level policies were primarily issued by the State Council and central health authorities, whereas local-level policies were drawn from provincial and sub-provincial governments and their health administrative departments.

Third, temporal representativeness was ensured by selecting policies distributed across different stages of policy development from 2018 to 2025, thereby capturing the evolution of institutional arrangements over time.

Fourth, only policy documents with clear structure, substantive content, and sufficient information to support multi-dimensional PMC evaluation were included. Policies lacking explicit policy instruments, implementation mechanisms, or evaluative components were excluded to ensure the reliability of indicator coding and scoring.

Fifth, priority was given to policies that reflect key institutional arrangements, major implementation pathways, and core governance mechanisms, in order to capture the essential features of policy design within the overall policy system.

Based on these criteria, a total of 24 policy documents were selected as representative samples, including 12 national-level policies (**Table S10**) and 12 local-level policies (**Table S11**). The selected samples collectively reflect the diversity of policy types, governance levels, and temporal stages, thereby ensuring the analytical representativeness and methodological robustness of the PMC evaluation.This sampling approach is consistent with prior applications of the PMC index model, which typically focus on representative and information-rich policy texts rather than the full sample.

**Table S10.** National-Level PMC Sample.

| NO. | Policy Document | Issuing Date | Issuing Department |
| --- | --- | --- | --- |
| N1 | Opinions of the General Office of the State Council on Promoting the Development of "Internet Plus Healthcare" | 2018.04 | General Office of the State Council |
| N2 | Guiding Opinions on Promoting the Reform and Development of the Nursing Service Industry | 2018.07 | National Health Commission |
| N3 | Administrative Measures for Internet-based Diagnosis and Treatment (Interim) | 2018.07 | National Health Commission |
| N4 | Notice of the General Office of the National Health Commission on Carrying Out the Pilot Work of "Internet Plus Nursing Services" | 2019.01 | National Health Commission |
| N5 | Guiding Opinions of the National Healthcare Security Administration on Improving the Pricing and Health Insurance Payment Policies for "Internet Plus" Medical Services | 2019.08 | National Healthcare Security Administration |
| N6 | Notice of the General Office of the National Health Commission on Further Promoting the Pilot Work of "Internet Plus Nursing Services" | 2020.12 | National Health Commission |
| N7 | Notice on Further Advancing the "Five Ones" Service Action for "Internet Plus Healthcare" | 2020.12 | National Health Commission |
| N8 | Notice of the National Health Commission on Issuing the National Nursing Development Plan (2021-2025) | 2021.05 | Department of Medical Administration |
| N9 | Notice of the General Office of the State Council on Issuing the 14th Five-Year Plan for Universal Healthcare Security | 2021.09 | General Office of the State Council |
| N10 | Guiding Opinions on Further Advancing the Integrated Development of Medical and Elderly Care Services | 2022.07 | National Health Commission and 11 other relevant authorities |
| N11 | Notice on Issuing the Action Plan for Further Improving Nursing Services (2023-2025) | 2023.06 | National Health Commission and National Administration of Traditional Chinese Medicine |
| N12 | Notice on Issuing the Action Plan for Enhancing Geriatric Nursing Service Capacity | 2025.12 | National Health Commission and 3 other relevant authorities |

**Table S11.** Local-Level PMC Sample.

| NO. | Policy Document | Issuing Date | Issuing Department |
| --- | --- | --- | --- |
| L1 | **Notice of Beijing Municipal Health Commission, Beijing Municipal Market Supervision Administration and Beijing Municipal Healthcare Security Administration on Developing and Regulating Internet-based Home Care Services (Jing Wei Yi [2018] No. 214)** | 2018.12 | **Beijing Municipal Health Commission, Beijing Municipal Market Supervision Administration and Beijing Municipal Healthcare Security Administration** |
| L2 | **Work Plan of Beijing Municipality for the Pilot Program of Geriatric Medical and Nursing Services** | 2022.09 | Beijing Municipal Health Commission |
| L3 | **Implementation Plan for the Pilot Work of "Internet Plus Nursing Services" in Tianjin** | 2019.04 | **Tianjin Municipal Health Commission** |
| L4 | **Action Plan for Further Improving Medical and Nursing Services in Tianjin (2023-2025)** | 2023.07 | **Tianjin Municipal Health Commission** |
| L5 | **Notice on Issuing the Implementation Plan for the Pilot Work of "Internet Plus Nursing Services" in Shanghai** | 2019.07 | **Shanghai Municipal Health Commission** |
| L6 | **Notice of Shanghai Municipal Civil Affairs Bureau on Issuing the Shanghai Three-Year Action Plan for Promoting the Construction of Smart Senior Care Homes (2023-2025)** | 2022.12 | **Shanghai Municipal Civil Affairs Bureau** |
| L7 | **Notice on Issuing the Implementation Plan for the Pilot Work of "Internet Plus Nursing Services" in Jiangsu Province** | 2019.04 | **Jiangsu Provincial Health Commission** |
| L8 | **Several Measures on Deepening the Integration of Medical and Elderly Care Services** | 2020.11 | **Jiangsu Provincial Health Commission** |
| L9 | **Notice of Zhejiang Provincial Health Commission on Issuing the Implementation Plan for the Pilot Work of "Internet Plus Nursing Services" in Zhejiang Province (Interim)** | 2019.05 | **Zhejiang Provincial Health Commission** |
| L10 | **Notice of Zhejiang Provincial Health Commission on Issuing the Implementation Plan for Further Promoting the Application of "Zheli Nursing" in Zhejiang Province** | 2022.12 | **Zhejiang Provincial Health Commission** |
| L11 | **Notice on Issuing the Implementation Plan for the Pilot Work of "Internet Plus Nursing Services" in Guangdong Province** | 2019.04 | **Guangdong Provincial Health Commission** |
| L12 | **Notice on Issuing the Pilot Work Plan for Geriatric Medical and Nursing Services in Guangdong Province** | 2022.04 | **Guangdong Provincial Health Commission** |

# Radar chart analysis

**Figure S1** and **Figure S2** visually summarize the dimensional profiles of PMC performance at the national and local levels. The national level radar profile is generally more extended and balanced, suggesting comparatively stronger structural completeness and design coherence. The local level profile, by contrast, appears less balanced, with more evident inward contraction in several dimensions. In particular, policy effectiveness shows the most visible weakness at the local level, while policy evaluation also remains relatively constrained at both levels. These visual differences are consistent with the main text findings that national policies achieved higher overall PMC performance, whereas local policies showed more pronounced structural imbalance across dimensions.

**Figure S1.** Comparative Radar Profiles of PMC Dimensions at National Levels.

**
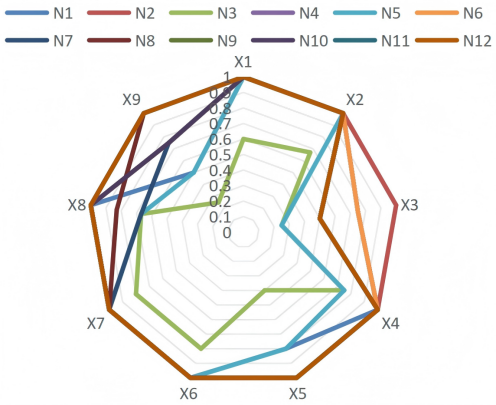
**

**Figure S2.** Comparative Radar Profiles of PMC Dimensions at Local Levels.

**
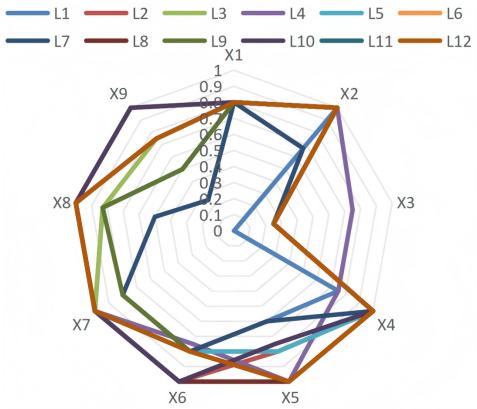
**
